# Environmental DNA reveals arboreal cityscapes at the Ancient Maya Center of Tikal

**DOI:** 10.1038/s41598-021-91620-6

**Published:** 2021-06-16

**Authors:** David L. Lentz, Trinity L. Hamilton, Nicholas P. Dunning, Eric J. Tepe, Vernon L. Scarborough, Stephanie A. Meyers, Liwy Grazioso, Alison A. Weiss

**Affiliations:** 1 Department of Biological Sciences, University of Cincinnati, Cincinnati, OH 45221 USA; 2 Department of Plant and Microbial Biology and the BioTechnology Institute, University of Minnesota, St. Paul, MN 55108 USA; 3 Department of Geography and GIS, University of Cincinnati, Cincinnati, OH 45221 USA; 4 Department of Anthropology, University of Cincinnati, Cincinnati, OH 45221 USA; 5 Department of Archaeology, Universidad de San Carlos de Guatemala, Ciudad Universitaria, 01012 Guatemala, Guatemala; 6 Department of Molecular Genetics, Biochemistry and Microbiology, University of Cincinnati, Cincinnati, OH 45267 USA

**Keywords:** Environmental impact, Plant sciences, Environmental sciences, Environmental social sciences

## Abstract

Tikal, a major city of the ancient Maya world, has been the focus of archaeological research for over a century, yet the interactions between the Maya and the surrounding Neotropical forests remain largely enigmatic. This study aimed to help fill that void by using a powerful new technology, environmental DNA analysis, that enabled us to characterize the site core vegetation growing in association with the artificial reservoirs that provided the city water supply. Because the area has no permanent water sources, such as lakes or rivers, these reservoirs were key to the survival of the city, especially during the population expansion of the Classic period (250–850 CE). In the absence of specific evidence, the nature of the vegetation surrounding the reservoirs has been the subject of scientific hypotheses and artistic renderings for decades. To address these hypotheses we captured homologous sequences of vascular plant DNA extracted from reservoir sediments by using a targeted enrichment approach involving 120-bp genetic probes. Our samples encompassed the time before, during and after the occupation of Tikal (1000 BCE–900 CE). Results indicate that the banks of the ancient reservoirs were primarily fringed with native tropical forest vegetation rather than domesticated species during the Maya occupation.

## Introduction

The focus of this study was to evaluate ways that the ancient Maya of Tikal managed the vegetation surrounding their major reservoirs to stabilize the embankments, provide ecological services and fulfill other sociocultural needs. Because the land management strategies that supported the development of complex societies in southern Mesoamerica overall are poorly understood, it is the objective of this research to elucidate how essential resources, such as water and forest products, were managed in complementary ways. More specifically, we are interested in the development of hydraulic systems in the Tikal polity as they provided an essential resource to an expanding community that grew exponentially during the Late Classic period (600–850 CE) without access to permanent water sources, such as rivers or lakes.

Up until the last decade, archaeological research at Tikal has largely concentrated on the architectural features, settlement patterns, ceramics, lithics, and other artifact assemblages. In stark contrast to the vast knowledge of material culture from the site, much less is known about the agricultural techniques, water management and ancient agroforestry practices employed by the Maya inhabitants. It has been the expressed purpose of our studies at Tikal to fill this gap in understanding, develop a more complete knowledge of the basic economic underpinnings of one of the great Maya polities and elucidate how its support system sustained the demographic, structural and political expansion of the Classic Period (250–900 CE). From the third century to the mid-ninth century CE the number of occupants at Tikal quadrupled^1^. At its zenith around 830 CE the population reached somewhere between 40,000 and 62,000 inhabitants^1–3^.

**Figure 1:**
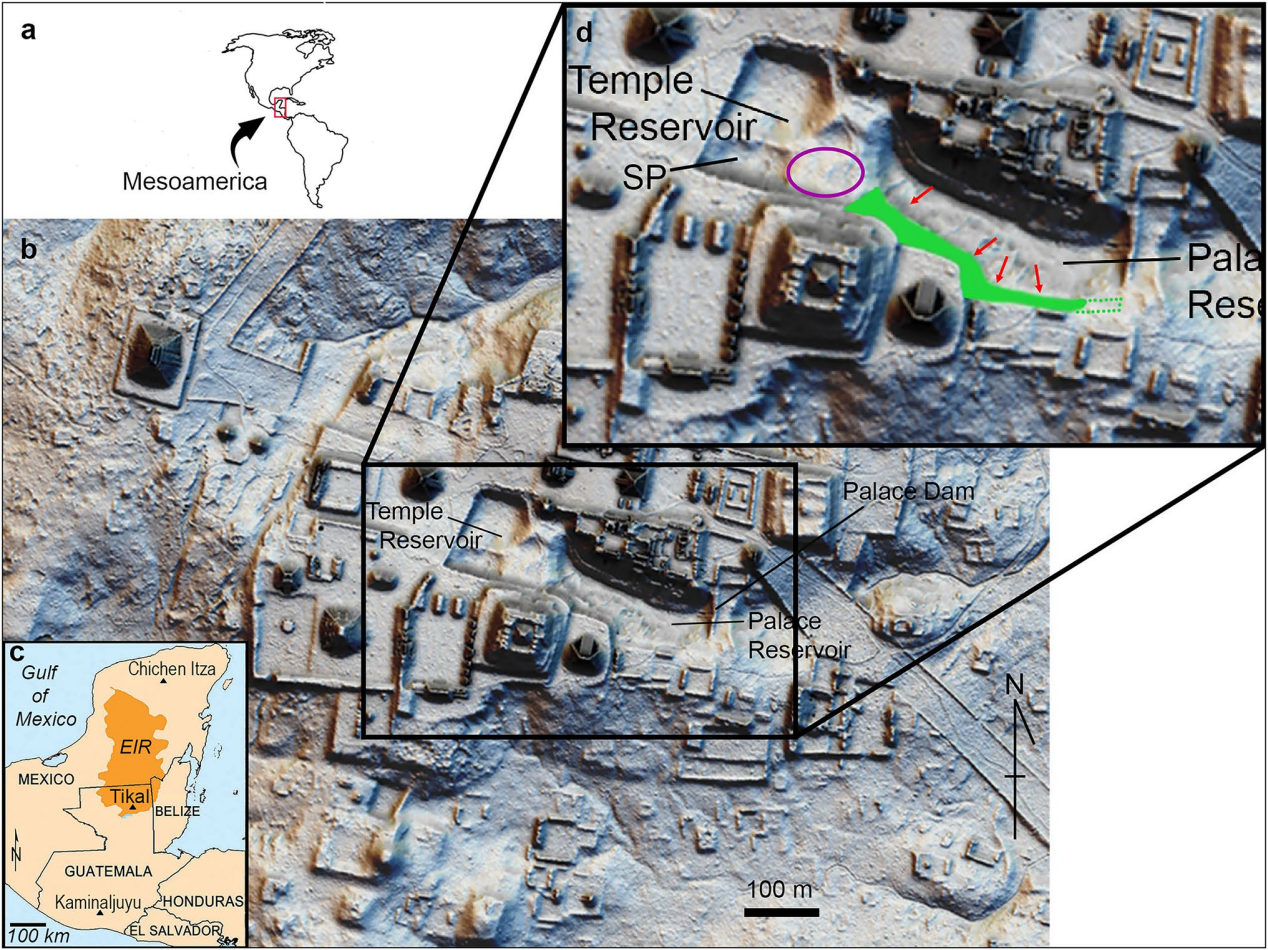
Lidar images of Tikal showing the location of the Palace and Temple reservoirs within the central precinct of the city. Providing water for large urban populations in this region was a challenge because there were no permanent lakes or rivers and the geological under-bedding is porous calcium carbonate. The map of the Western Hemisphere (**a**) shows the location of the Yucatan peninsula. The inset map (**c**) indicates the location of Tikal within the Yucatan peninsula and the extent of the elevated interior region (EIR). The central lidar image (**b**) presents a view of the site core of Tikal. The enlargement in the upper right corner (**d**) shows the water’s edge of the Palace reservoir (red arrows) and the location of the “bench” or vegetated embankment (green) discussed in the text. The location of the hypothesized “ancestral grove” is encircled in purple. The proposed silting tank or spring pool (SP) lies just to the west of the ancestral grove. The lidar image of the Tikal site core was courtesy of Francisco Estrada-Belli, MARI GIS Lab and the Fundación Patrimonio Cultural y Natural Maya-PACUNAM. Nicholas Dunning created the Mesoamerican inset map. David Lentz created the map of the Western Hemisphere and the final figure using Photoshop CS6, Vers. 13.0.1 × 64 (https://www.adobe.com/products/photoshop/pricing-info.html).

During excavations in 2009 and 2010 our team examined many aspects of land use, forest management and water control at Tikal^4^. We learned that the Tikal Maya built one of the most sizable earthen and stone dams in the Precolumbian Americas to create the Palace Reservoir, the centerpiece of the Temple-Palace-Hidden Reservoir chain^4,5^. Originating at a natural spring that over time was walled in on three sides, the spring pool dropped water into the Temple Reservoir (Fig. 1). This reservoir was also fed from runoff coming from surrounding paved areas and was separated from the topographically lower Palace Reservoir by a natural ridge and a narrow coffer dam. The Palace Reservoir was formed by the massive Palace Reservoir dam, which separated it from Hidden Reservoir, itself created by a causeway dam.

The Maya also devised an irrigation system that was fed by runoff from a largely paved ceremonial precinct and stored it in a distant water tank, the Perdido Reservoir, for release when needed^5,6^. Evidently, the Maya farmed the margins of the seasonal swamps (*bajos*) with maize (*Zea mays* L.) and root crops^7–11^ and built a unique filtration system for one of the reservoirs (Corriental) that involved the use of imported sand and zeolite^12^. Furthermore, we discovered that at least part of the reservoir system was plagued by chronic mercury contamination and periodic toxic blue-green algal blooms^13^. These developments exacerbated the impact of the Terminal Classic (850–900 CE) droughts^14–21^ that coincided with the abandonment of the site core (ca. 870 CE).

One problem the Maya did not seem to have with their Temple-Palace reservoir system was that of excessive erosion from surrounding earthen embankments. Even though the banks were quite steep in some cases, the build-up of sediment into the reservoirs was generally not a serious problem, at most it was a challenge resolvable by periodic draining and dredging^5^. This lack of sedimentation caused by upstream erosion was not surprising given that easily 90% of the catchment (approximately 30 ha) for these inter-connected reservoirs in the city center consisted of plastered structures and plazas. There were physical barriers that surrounded the reservoirs, but recently obtained lidar images of the Tikal site core revealed that there was a prominent bench, about 10–30 m wide, particularly on the south side of the Palace Reservoir, between what would have been the water’s edge and the beginning of the structural containment. We surmised that the Maya relied on vegetation along this bench to hold the soil in place and stabilize the watershed.

Following this conceptualization, we developed four hypotheses that could be tested using environmental DNA (eDNA) data extracted from reservoir sediments to determine what kind of plants were growing in the vicinity of these central reservoirs during the Maya occupation of Tikal. This technique was especially useful because many of the markers we used in the creation of our genetic probeset were from plastid (choloroplast) genes, which are over-represented in most plant tissues relative to nuclear genes, so leaves falling into the reservoirs could be detected. The two most likely areas that were vegetated in close proximity to the reservoir pools and from which plant material fell and accumulated were the bench of land just above the normal pool level in the Palace Reservoir and a ridge of higher land separating the Palace and Temple Reservoirs (Fig. 1). The bench covers an area approximately 150 × 30 m, and the ridge approximately 60 × 70 m. Because our probes focus on chloroplast DNA and because the Temple and Palace Reservoirs are higher in elevation than the surrounding area, outside of its very small watershed^13^, we posit that the vast preponderance of the DNA we recovered was from leaves that fell from plants adjacent to the reservoirs. Furthermore, ethnographic evidence^22^ tells us that the Maya have strict social taboos about contaminating ponds (*aguadas*) used for drinking water, so it is unlikely that the Maya rulers would have permitted the introduction of extraneous leaf detritus into the reservoirs that supplied their drinking water. The first hypothesis was that they planted the banks of their reservoirs with annual crop plants, such as maize (*Zea mays* L.), beans (*Phaseolus* sp.), and squash (*Cucurbita* spp.). The soils around the reservoirs likely were extremely fertile and would have produced excellent harvests. The time between harvesting and planting, however, would have exposed the fragile topsoil layer. Even if they had used a ridge and furrow system as seen elsewhere in the Maya world^23^ or other kinds of microtopographic soil conservation methods, it is difficult to imagine that this would have been an effective approach to embankment stabilization. In support of this hypothesis, however, Cortés, in early Post-Conquest times, observed that the shores of Lake Petén Itzá were lined with “*labranzas*” or tilled fields, that were seeded with various crop plants such as cotton^24^.

Because of the steepness of the slopes, the Tikal Maya probably employed some arrangement of perennial plants with extensive root systems that would hold the soil tightly and prevent any kind of percussive effects from falling rain on exposed soil. A second hypothesis, once again looking at the fertility of the soil adjacent to the reservoirs, argues that the ancient Maya planted fruit trees such as avocado (*Persea americana* Mill.), anona (*Annona* spp.), cacao (*Theobroma cacao* L.) or any of the other orchard crops that have been previously identified at Tikal using standard paleoethnobotanical techniques (*SI Appendix*; Table S1). Orchards for this purpose could have been intercropped with a host of perennial shrubs that were common at Tikal. Because of the large number of domesticated tree species found among the archaeological plant remains^7^, we know that theTikal Maya were planting orchards somewhere in the vicinity of the site. By way of comparison, the huge Preclassic Purrón Reservoir in southern Mexico, probably the largest in ancient Mesoamerica, was surrounded by orchards^25^. Orchard plantings have been hypothesized for plots near the elite residential district at Cobá, another major Maya center^26^. Similarly, “ancestral orchards” adjacent to royal residences were believed to have been a hallmark of dynastic inheritance in the Maya area^27^. Note that the Palace and Temple reservoirs had elite residential structures immediately to the north and south, so these artificial lakes were likely appreciated for their scenic appeal as well as for their practical function of providing clean water. This kind of planting arrangement, with orchards surrounding the reservoirs, would have been a highly effective system for curbing erosion, creating a zone of cool and well-shaded repose and enhancing the overall agricultural productivity of the city.

A third hypothesis was that the Maya allowed the embankments to remain as undisturbed forest or possibly planted forest trees where needed, similar to the Postclassic and modern Mexican farmers of Xochimilco who planted native willow and cypress trees to stabilize the banks of canals^28^. The tree species in the tropical deciduous forest of the northern Petén are adapted to the rainy season downpours and their root systems hold the soil remarkably well. Moreover, many forest species produce edible fruits, medicinal secondary compounds and are useful in other ways, providing numerous reasons why this would have been a viable option.

A fourth hypothesis posits the use of other kinds of native vegetative growth in and around the reservoirs. These include waterlilies (*Nymphaea* spp.) which were highly symbolic plants for the ancient Maya and may have reduced evaporation from reservoir surfaces, especially in the hot dry seasons^29^. Waterlilies are often illustrated on Maya wall paintings^30^ and other kinds of artwork^31,32^. Also, rulers of Tikal have been referred to as “people of the reeds” or *Ah Puh*^31^. The reeds in this reference were most likely cattails (*Typha* spp.) and would have grown well in the areas adjacent to the springs and, subsequently, the perimeters of the reservoirs. The primary purpose of this study was to test and evaluate all of these hypotheses using cutting edge eDNA technologies.

## Results

DNA extraction was performed on 30 sediment samples from Tikal reservoirs and aguadas (both natural and human altered karstic depressions). Eukaryotic DNA was obtained from four archaeologically meaningful reservoir samples and one modern control sample. These provided the essential data source discussed in this paper. Variable sequences from plastid and nuclear genes of Mesoamerican plants were provided by the authors of this paper to RAPiD Genomics LLC (Gainesville, FL). This genetic dataset enabled them to synthesize 120-bp probes used to capture homologous sequences from ancient DNA extracts through their Capture-Seq protocol. The sediment DNA was amplified, sequenced, and assembled into contigs, or overlapping DNA segments, that together represented a consensus region of DNA.

The contigs, or nodes, were individually subjected to analysis using the Basic Local Alignment Search Tool (BLAST) algorithm that compared our archaeological DNA sequences to the entire GenBank nucleotide database of the National Center for Biotechnology Information (NCBI). This BLAST analysis allowed us to identify homologous sequences from the database (Tables S2–S5). The plastid probes in particular yielded plant-specific hits. Thirty-seven sequences from the reservoir sediments could be assigned to a single genus, with different species often having nearly equivalent homology scores. Species assignments were made to plant gene sequences with the highest Bitscore values and known to be native to Guatemala^33,34^. Due to high sequence conservation, BLAST hits to some of the plant contigs could only be resolved to genus, family or order taxonomic levels.

Of the four ancient DNA samples selected for sequencing, one was from the Temple Reservoir and three were from the Palace Reservoir. Strata containing these samples were previously dated by AMS ^14^C analysis (Table S6) from the Early Preclassic period (1780–1620 BCE) to the Early Postclassic period (ca. 900–1100 CE), encompassing over 2500 years, including times before, during, and after the Maya occupation of the Tikal site core. Results of the eDNA analysis are listed in Table 1 and will be discussed in chronological order, beginning with the earliest.

**Table 1:**
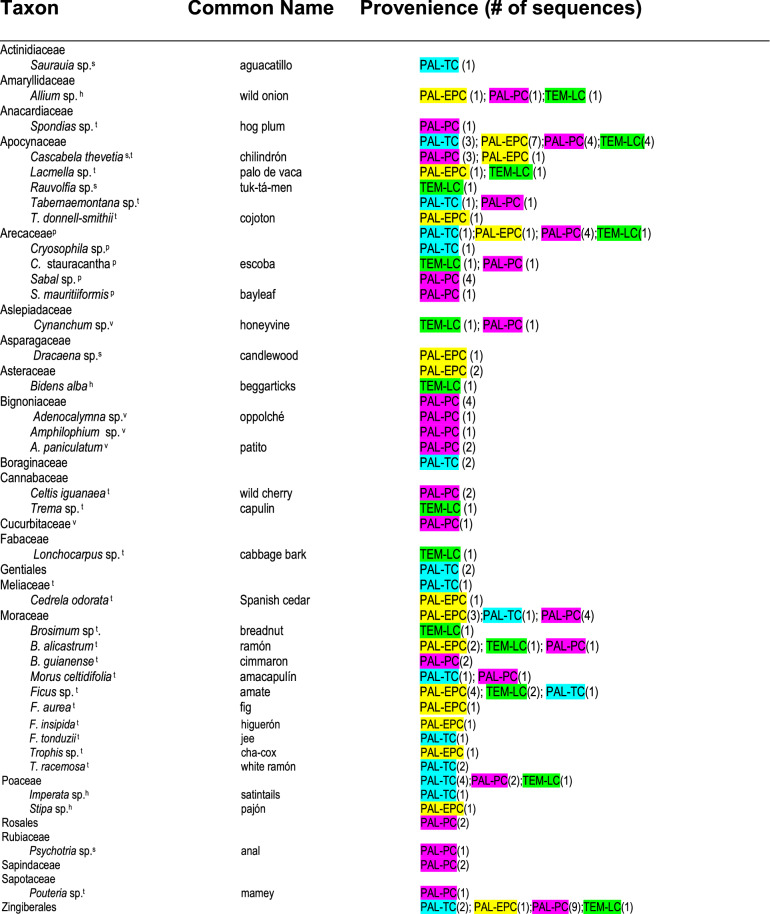
Environmental DNA results from Temple and Palace reservoirs at Tikal.

Palace, Early Preclassic period =  (WA07); Temple, Late Classic period =  (WA09); Palace, Terminal Classic period =  (WA01); Palace, Postclassic period =  (WA08). Growth habit: *t* tree, *s* shrub, *h* herb, *v* vine, *p* palm.

**Figure 2:**
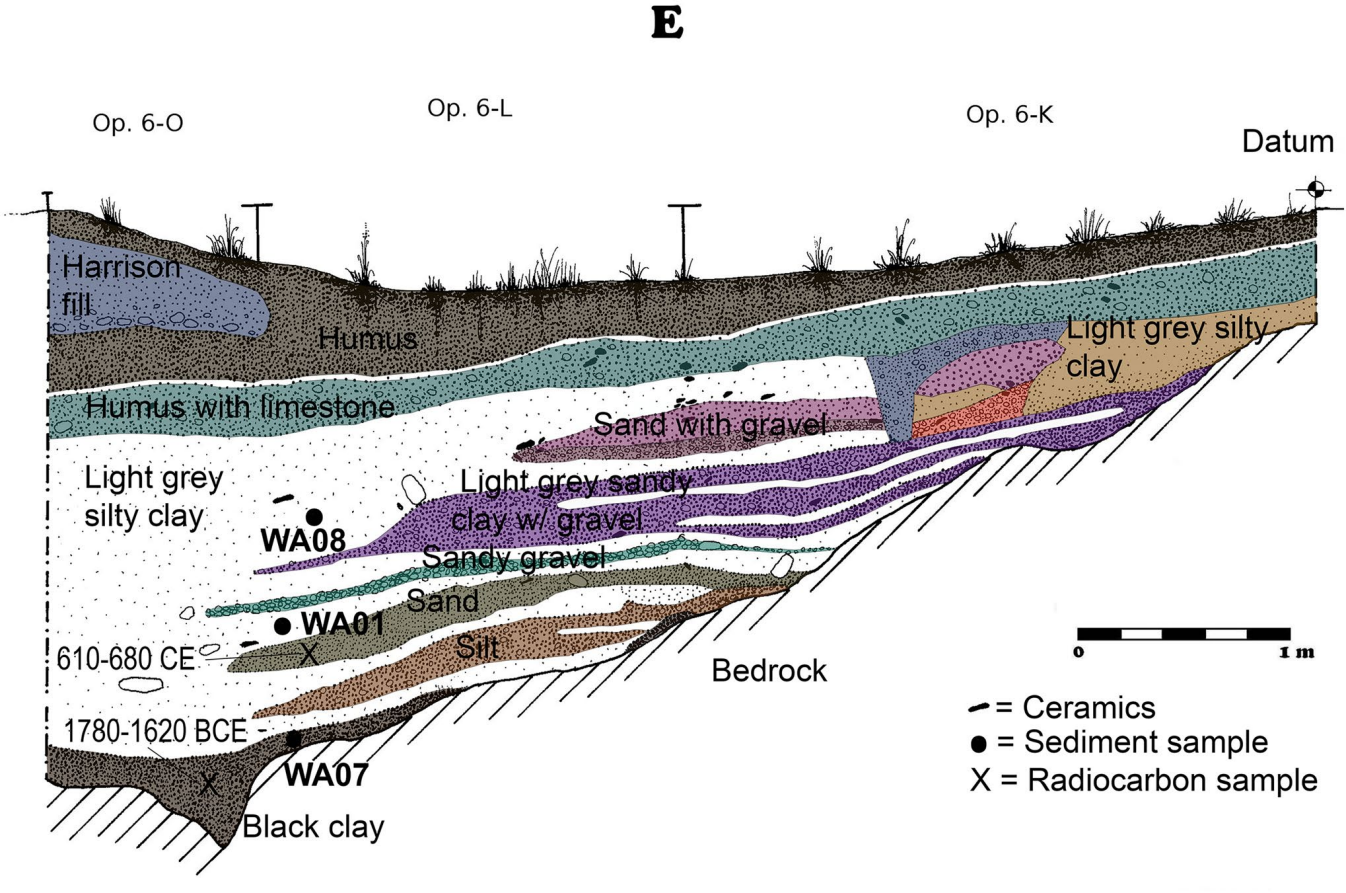
Stratigraphic profile of Palace Reservoir excavations showing Operations K, L and O. Collection locations for samples WA01, WA07 and WA08 are indicated on the profile as well as relevant AMS radiocarbon (^14^C) dates. The original drawing was prepared by Brian Lane. David Lentz created the final figure using Photoshop CS6, Vers. 13.0.1 × 64 (https://www.adobe.com/products/photoshop/pricing-info.html).

The Early Preclassic sample (WA07) was collected from a black clay layer that lies just above bedrock and below the bottom of what became the Palace reservoir (Fig. 2). It predates the known Maya occupation of the site and the construction of the Palace Dam. Accordingly, this sample reveals the kind of vegetation that blanketed the ravine (that was eventually converted into a reservoir) in a relatively undisturbed state. In that sample, there were large forest trees represented (*Brosimum alicastrum* Sw. and *Cedrela odorata* L.), understory trees (*Tabernaemontana donell-smithii* Rose, *Trophis* sp. and *Ficus* spp.), shrubs (*Dracaena* sp. and *Cascabela thevetia* [L.] Lippold), and herbaceous plants (*Allium* sp. and *Stipa* sp.).

The Late Classic sample (WA09) from Temple Reservoir sediments, contained DNA from large trees *Brosimum alicastrum* and *Lonchocarpus* sp., understory trees *Ficus* sp. and *Lacmellea* sp., a shrub *Rauvolfia* sp., a vine *Cynanchum* sp., a small palm *Cyrosophila staurocantha* (Heynh.) R.J. Evans and an herbaceous plant, *Allium* sp. This assortment of plants is generally typical of an upland tropical forest. Otherwise, there were two taxa typical of disturbed habitats represented in this sample, *Bidens alba* (L.) DC. and *Trema* sp. The former is a weedy composite and the latter is a genus of small trees. This sample reflects a time period when the population and the cultural inflorescence of Tikal was at its apogee.

The Terminal Classic sample (WA01, 850–900 CE) from the Palace Reservoir reflects a time that was close to the abandonment of Tikal. There were no big trees represented other than a single gene sequence identified as Meliaceae, a family of a few large trees (e.g., *Swietenia macrophylla* King, *Guarea* spp., and *Cedrela odorata*) and a large genus of small trees, *Trichilia* spp. There were other understory trees, too, identified in this sample, viz., *Trophis racemosa* (L.) Urb., *Ficus tonduzii* Standl.*, Morus celtidifolia* Kunth and *Tabernaemontana* sp. The diminutive understory palm, *Cryosophila stauracantha*, also was present. An attractive understory shrub that bears edible fruit, *Saurauia* sp., was indicated in this sample, as well. All of these species are common in riparian habitats and other wet places^35–37^ and would have thrived in the damp environs of the littoral zone around the reservoir. Finally, evidence for *Imperata* sp., a genus of tough, perennial, tropical grasses, also was identified from this sample.

WA08, the Postclassic period (900–1100 CE) sample from the Palace Reservoir, represented a time following the abandonment of the city. There are several large trees identified including *Brosimum alicastrum* Sw., *Pouteria* sp. and the large palm, *Sabal mauritiformis* (H. Karst) Griseb. & H. Wendl. Understory trees included *Spondias* sp*.*, *Celtis iguanea* (Jacq.) Sarg., *Morus celtidifolia* Kunth. and *Tabernaemontana* sp. Two shrubs, viz., *Psychotria* sp. and *Cascabela thevetia* (L.) Lippold, several vines, (*Adenocalymma* sp., *Amphilophium paniculatum* [L.] Kunth, and a plant in the Cucurbitaceae) and one wild herbaceous plant, *Allium* sp., also were detected from this context. None of our analyses discussed in this paper have revealed evidence for waterlilies or cattails.

**Figure 3:**
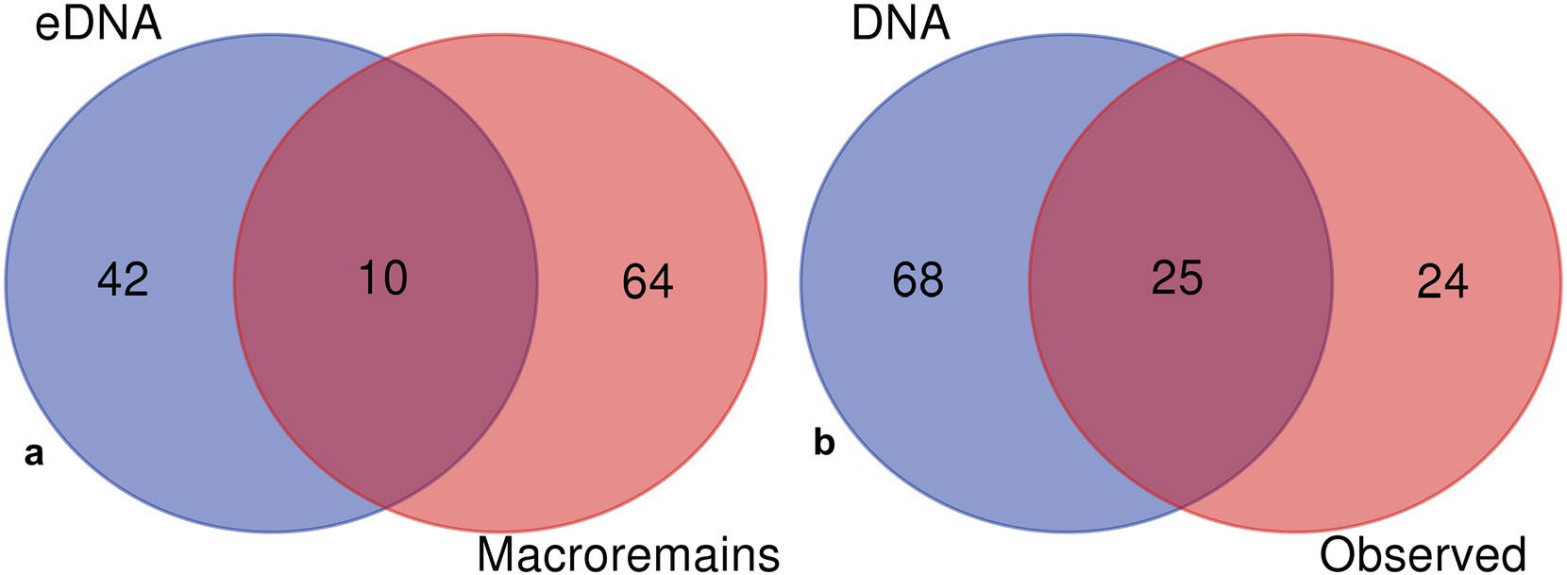
Diagrams showing: (**a**) the overlap in archeological plants identified at Tikal using eDNA technologies compared to conventional paleoethnobotanical analyses of plant macroremains and pollen and (**b**) the overlap between the results of soil DNA analyzed from a household garden in the village of Uaxactun, Guatemala, as compared to plants actually observed in the garden. David Lentz created the final figure using Photoshop CS6, Vers. 13.0.1 × 64 (https://www.adobe.com/products/photoshop/pricing-info.html).

A fifth DNA sample was collected from the nearby modern village of Uaxactun. A list of all of the major plants observed in a home garden, or *huerta,* of the village was recorded and a surface soil sample was collected from the middle of the garden. The soil sample (WA05) was processed using the same protocols for extraction and sequencing that were used for the ancient Tikal samples. We performed this exercise to demonstrate the efficacy of the techniques applied to the archaeological samples. 93 taxa were identified from the Uaxactun garden DNA sample, while 49 species were observed during a physical inspection of the garden. Over half of the plants, 25, actually observed within the garden (Table S7) were also detected by the DNA results (Fig. 3; Table S8). Plants that were physically observed in the garden and detected from the DNA sampling (Table S7) included most of the major fruit trees, medicinal plants, ornamentals and crop plants that appeared in the garden.

## Discussion

A number of salient observations can be readily discerned from these results. First, of our three hypotheses regarding the vegetation surrounding the Palace and Temple reservoirs in ancient times, the third hypothesis, that the ancient Maya maintained native forest plants on the banks of the reservoirs is strongly supported. This is a welcome insight into the vegetation surrounding Maya center-city reservoirs. If we look closely at the eDNA results, there are nuanced indications from the data recovered, as well. To begin, there is no clear evidence for the presence of cultigens. From the Postclassic sample (WA08), there is a genetic sequence from the Cucurbitaceae, which includes the squashes and pumpkins, but there also are many wild species in the family that are native to the Neotropics, so results in this case were inconclusive. The same is true with the Poaceae sequences; maize is not clearly indicated in any of the reservoir samples and the only two grasses that were positively identified were *Stipa* sp. and *Imperata* sp., both wild genera native to the region. In summary, these observations support the validity of hypothesis three, i.e., the Maya did not plant cultigens around these two reservoirs, but allowed or encouraged native forest species to grow on the banks.

These findings suggest that in some cases trees and plant vegetation were near the banks of these reservoirs for other reasons, too. Given the central location of the two reservoirs at issue, they may have been used as recreational settings or aesthetic zones within the proximity of numerous towering architectural structures. The functionality of the reservoirs is clear—they were sources of potable water—but trees for shading, understory cooling and symbolic effects may have been an important concern, as well^38^.

The earliest sample (WA07 from the Early Preclassic period) and most recent sample (WA08 from the Postclassic period) in a way represent bookends of the Maya occupation, with the earlier sample pre-dating the Maya occupation of the site and the latter sample representing a time period following the ninth century droughts and the abandonment of the site core of the city. They reflect similar environments with tall forest dominants, understory trees, vines and shrubs suggestive of well-structured, multi-tiered, semi-deciduous tropical forests, typical of the northern Petén region^39^.

The two middle samples, however, present slightly different pictures. The Late Classic period sample (WA09) has some forest dominants, *Brosimum alicastrum* and *Lonchocarpus* sp. and some understory trees, but there are also some disturbance taxa appearing, such as *Bidens alba,* which is a common weed^40^ and *Trema* sp. which is a genus of small trees generally found in second growth^40^. The presence of these invaders suggest habitat disturbance and that the forest canopy around the reservoir may have been opening up.

A question arises in regard to the results from the Temple Reservoir. On close inspection of the lidar image (Fig. 1), there is really no appreciable space around the reservoir where an embankment may have been positioned and it is, for the most part, surrounded by pavement. There is, however, a large zone to the east and the southeast of Temple Reservoir, a low ridge where there appears to be an undisturbed land surface (approximately 400 m^2^) adjacent to what has been referred to as a “silting tank”^5^ where the freshwater spring was found. Another, more cosmological interpretation^38^, suggests that this area may have been a sacred well for the city, or *ch’en,* and the undisturbed land adjacent to it may have supported an ancestral grove of trees^38^ associated with the sacred well. One curiosity of the Temple Reservoir sample was the indication of *Brosimum alicastrum*, or *ramón*. It is one of the oligarchic species of the Neotropical forest and it can grow to be a huge tree with massive roots. This is not a tree that you would plant next to your reservoir because the roots could disrupt the floor of the plastered reservoirs. This argues for the presence of this tree on the ridge where it would have been least disruptive to the integrity of the plastered reservoirs. Also, the *ramón* find argues for this representing part of an ancestral grove that the Maya built around when creating the reservoir system.

Another curiosity concerning the *ramón* discovery from the Temple Reservoir relates to an earlier hypothesis that ramón fruits were an important foodstuff for the ancient Maya^41^. While this seems like a logical assertion because the fruits are edible and were certainly abundant during ancient Maya times, analysis of thousands of archaeobotanical specimens from Tikal produced very little evidence to substantiate this idea^7,10^. While the Tikal Maya may have eaten *ramón* fruits during times of scarcity, it is unlikely that it was an important component of their regular diet.

During the Terminal Classic period, the results of sample WA01 offer no clear evidence for the presence of dominant forest tree species in the vicinity of the Palace Reservoir. There is the Meliaceae sequence, but it is unresolved whether this represents a large tree, e.g., *Cedrela odorata*, or one of the smaller understory species, such as *Trichilia* spp. Because the large Meliaceae tree species are important commercially, Genbank has numerous DNA sequences in the database from these trees. Alternatively, trees in the genus *Trichilia* are poorly represented so it may be more likely that one of the smaller species is represented by the Meliaceae sequence. Several other small trees were clearly identified in this sample, viz., *Ficus tonduzii*, *Trophis racemosa*, *Tabernaemontana* sp., *Morus celtidifolia* and the diminutive palm, *Cryosophila stauracantha*, would have made an attractive and effective lining along the littoral zone of the south bank of the Palace Reservoir. Finally, *Imperata* sp., a perennial grass of wastelands and forest transition areas^40^ makes an appearance during this time period. These plants probably took hold around the reservoir edges where there was more light available. The droughts of the mid-ninth century may have opened up more habitat for this aggressive grass. Quite possibly, as the water levels in the reservoirs receded due to the droughts, the *Imperata* grass could have followed the waterline downward as the native trees, vines and shrubs suffered. Looking at the eDNA results as a whole, it appears evident that the Maya favored the maintenance of native vegetation on the banks surrounding the reservoirs and their erosion control strategy worked well for many centuries.

The second noteworthy aspect of this research relates to the Uaxactun garden experiment. As mentioned above, there was a substantial overlap of 25 plants between those plants physically observed in the garden (total 49) and those detected by the genetic probe (total 93). Although the overlap was not complete, the 25 plants that were recorded by both methods were the trees, shrubs, herbs and crops of most economic value^36^. *Brosimum alicastrum* was detected by the DNA probe but not observed in the garden, a common forest dominant in the region^42,43^. The leaves and fruits of *ramón* were likely brought into the compound. As mentioned above, the fruits of *ramón* are edible and the leaves are commonly used for livestock fodder^36^. Accordingly, the DNA probe detected not only the plants growing in the garden at the time, but also plants brought in from the surrounding forests and likely from neighbor’s gardens, as well. The results from the Uaxactun home garden analysis demonstrated two important features of our methodology: (1) that our probe could readily detect the presence of domesticated plants and (2) the probe detected the presence of sufficient plant data to effectively characterize the environment from where the sample was collected.

The third important aspect of the information gained from the eDNA analysis was that the procedures followed in this study are an important complement to other paleoethnobotanical techniques such as pollen, macro-remain, phytolith and starch grain analysis. When we compare the results from the traditional paleoethnobotanical methods to those of the eDNA analysis (Fig. 3, Tables 1 and S1), we see a distinct overlap, yet all approaches produced some unique species undetected by the other. Note that our efforts to extract pollen from the Temple and Palace reservoirs were unsuccessful and this underscores the value of the eDNA data. Thus, it appears that eDNA analysis can add significantly to the paleoethnobotanical toolkit and our findings will be much richer and more detailed if, in the future, all of the techniques mentioned above are used in combination.

Finally, perhaps the most important result from this study was that we were able to extract DNA from ancient Mesoamerican reservoir sediments, sequence the extracts and identify species-specific sequences. This achievement represents a technological leap forward for the field of archaeology. Other scholars working with ancient DNA have encountered issues with degradation of genetic material over time^44–46^ as we have in our study. Notwithstanding the challenges of preservation, it is clear that in some contexts, e.g., clayey strata, DNA can be preserved and recovered. Some researchers have stated that the eDNA approach has enormous potential to efficiently detect human environmental impacts through time^47^ while others have predicted that the technique will “revolutionize” the field of archaeology^48^. This study, although limited in scope, may help to place those predictions on a pathway to fruition.

## Materials and methods

Thorough descriptions of the materials and methods employed in this study, both in the field and in the laboratory, are presented in the Supporting Information section. Briefly, following collection of field samples in columns^49^ using sterile containers, sediment samples from Tikal reservoirs were hand carried to lab facilities in the USA. After arrival at the University of Cincinnati, samples designated for molecular genetic analysis were placed in a – 80 °C freezer until processing could begin. Just prior to DNA extraction, samples were thawed to 4 °C then inserted, under sterile conditions, into tubes with glass beads then sealed prior to homogenization in a bead-beating machine. To avoid the possibility of contamination, samples were processed in an isolated laboratory in the Department of Biological Sciences that previously had been dedicated to the extraction of DNA from bacteria, so there was no chance of contamination from vascular plant DNA, the target of our study. All standard protocols to detect and prevent incursion from contaminants were employed^50,51^. The clean bench was exposed to UV light when not in use and was cleaned with 70% ethanol and bleach between extractions following strict workflow procedures. DNA was extracted from sediment samples using DNeasy Power Soil Kits (Qiagen) as described previously^13,52,53^. To process the fragmented genetic material recovered from DNA extractions, we enlisted the services of RAPiD Genomics LLC (Gainesville, FL) to design genetic probes using their capture-Seq protocol to capture plant genes across a wide variety of taxa (Table S9). Target genetic markers, e.g., trnL, rbcL and ITS regions, similar to those used successfully in previous ancient vegetation studies^54–56^ were employed to construct the probes. Samples were pooled equimolar and sequenced using an Illumina HiSeq 2X150 sequencing system followed by bioinformatics processing.

## Supplementary Information

**Supplementary Information**.

## Data Availability

Raw sequencing reads are available on the NCBI Sequence Read Archive under accession numbers SAMN16484564 to SAMN16484570. Tikal project reports and profile drawings can be accessed from the Mesoweb website (http://www.mesoweb.com/es/informes/PSMAT-Lentz_et_al_2011.pdf).
