# Supplementary material for: Environmental DNA reveals arboreal cityscapes at the Ancient Maya Center of Tikal
Source: Sci Rep. 2021 Jun 16;11:12725. doi: 10.1038/s41598-021-91620-6 (PMC8209062; doi:10.1038/s41598-021-91620-6)
Supplement: Supplementary file 1 — Supplementary Information. [file 41598_2021_91620_MOESM1_ESM.pdf]

Supplementary Information

Environmental DNA Reveals Arboreal Cityscapes at the Ancient Maya Center of Tikal

David L. Lentz[1*], Trinity L. Hamilton[2], Nicholas P. Dunning[3], Eric J. Tepe[1], Vernon L. Scarborough[4], Stephanie A. Meyers[1], Liwy Grazioso[5], Alison A. Weiss[6*]

[1]Department of Biological Sciences, University of Cincinnati, Cincinnati, OH, 45221, USA. [2]Department of Plant and Microbial Biology and the BioTechnology Institute, University of Minnesota, St. Paul, MN, 55108, USA. [3]Department of Geography & GIS, University of Cincinnati, Cincinnati, OH, 45221, USA. [4]Department of Anthropology, University of Cincinnati, Cincinnati, OH, 45221, USA.[5]Department of Archaeology, University of San Carlos of Guatemala, Ciudad Universitaria, 01012, Guatemala. [6]Department of Molecular Genetics, Biochemistry and Microbiology, University of Cincinnati, Cincinnati, OH, 45267, USA. Correspondence and requests for materials should be addressed to D.L.L. or A.A.W. (*email lentzdl@ucmail.uc.edu, weissaa@ucmail.uc.edu)

SI: Archaeological Background

Within the Maya Lowlands lies an irregularly-shaped physiographic province known as the Elevated Interior Region (EIR; Fig.1), a karst area characterized by an acute lack of perennial surface water and almost no access to groundwater (1). Given a regional climate with a 5-month-long dry season, year-round occupation of the EIR by large numbers of Maya people and urbanization was dependent on their ability to capture and store enormous quantities of rain water during the rainy season. One of the first archaeological explorations of the northern part of the EIR by John Lloyd Stephens (and artist companion Frederick Catherwood) noted: “Among the wonders unfolded by the discovery of these ruined cities, what made the strongest impression on our minds was the fact that their immense population existed in a region so scantily supplied with water (2).” Catherwood illustrated several ancient reservoirs, including a hypothetical cross-section of one that had been cleaned out by an agricultural estate owner and included a number of chambers and wells constructed in the floor of the reservoir. Early explorers of the EIR, both scientific and economic, e.g., chicle harvesters and loggers, were utterly dependent on obtaining water during the dry season from ancient Maya reservoirs and were acutely aware of their association with ruined settlements (3-6). The 19th century reuse of ancient reservoirs was a common practice in the northern EIR (7).

The first modern archaeological excavations of ancient Maya reservoirs took place in the 1950s and 1960s as part of the University of Pennsylvania Tikal Project. Peter Harrison (8) published a short description of the Penn excavations in the Museum's journal *Expedition,* otherwise most of the Penn Project reservoir research remains unpublished.

Gary Gallopin and Vernon Scarborough used the University of Pennsylvania Tikal Project's detailed topographic and architectural maps to make a hydrological analysis of the reservoir system within central Tikal (9). Those analyses, along with the Lentz and Hockaday study (10) of Tikal's archaeological plant remains, formed the impetus for the creation of the University of Cincinnati Tikal Project which examined ancient Maya water, forest, and land use around Tikal. Excavations were conducted in 2009 and 2010 and laboratory analyses have been ongoing. Excavations were conducted in Palace, Temple, Hidden, Perdido, Corriental, and Pital reservoirs in and just south of the site center, as well as the Aguada de Terminos, Aguada Vaca del Monte, and Aguada Elmer in and around the Bajo de Santa Fe several kilometers east of the site center (11-14). Among the findings was the discovery that the feature known as the Silting Tank or ("Spring Pool") situated topographically above the Temple Reservoir was constructed around a natural spring, likely in the Mid- to Late Preclassic period (ca. 521-216 BCE).

The Palace Reservoir included a system for periodic maintenance and water-level adjustment by employing stacked sluice portals in the Late Classic dam which encased a smaller Early Classic dam. Particulate analysis of sediments from several of Tikal's reservoirs indicate that the region received episodic volcanic ash fall (15,16). Geochemistry and genetic analysis of several reservoirs revealed that Temple and Palace reservoirs were highly contaminated with toxic cyanobacteria and mercury as water levels declined during Terminal Classic droughts in the 9th century CE, whereas Corriental, Perdido, and Terminos reservoirs remained less affected (17).

The water complex that includes, in descending order, the Temple Reservoir and associated Spring Pool or Silting Tank, the Palace Reservoir, and the Hidden Reservoir, lies at the heart of Tikal surrounded by royal palaces and several major temple pyramids among other structures. The most elevated element in the complex is the so-called "silting tank," a name assigned by Scarborough and Gallopin (9) to a relatively small square tank perched above the Temple Reservoir and connected to it by a sluice. The assigned name was based on a model of water flow and sediment settling found in some other ancient Maya reservoirs within fluvial systems, e.g., at Kinal, Guatemala (18) and in other parts of the world. One problem with this interpretation at Tikal is that the "silting tank" is essentially perched at the head of a drainage that would have received very little fluvial input. Furthermore, almost all of its catchment would have been paved by the Early Classic period or earlier, hence, there was likely little sediment being deposited in this tank. Some sediment did accumulate beginning

in the Late Preclassic as well as in the Classic period, with some indication that dredging may have taken place (11,12). Also arguing against the “silting tank” functioning as its name implies was the discovery of a natural spring within this feature; it began to flow when one of our excavations breached a heavy cap of clayey sediment (11,12). Thus, we suggest that this feature was a Spring Pool built to contain the water of the spring, a place that would have had tremendous symbolic value to Tikal and its rulers. Water sources were closely linked to the places of foundation for ancient Maya dynasties (19).

The Spring Pool disgorged water into the subadjacent Temple Reservoir, which also received runoff from surrounding paved surfaces. Sediments within the Temple Reservoir tank, superimposed over a thick clay liner above limestone bedrock, dated exclusively to the Late Classic period, probably indicating that the tank was dredged episodically until sometime in the Late Classic (11-12, 20). The Temple Reservoir and Spring Pool were separated from the Palace Reservoir by a limestone bedrock ridge (Fig.1). Discharge from the Temple Reservoir flowed through a channel at its northern end into the Palace Reservoir and was controlled by a coffer dam.

The Palace Reservoir was created by the damming of a natural ravine initially by a small dam in the Early Classic period, but later by the massive Palace Reservoir dam, a huge construction of rock, masonry, and clay probably anchored by bedrock projections at its north and south ends (12). Within the reservoir some steps or benches were created by a combination of quarrying bedrock, flagstone paving, and earthen embankments. Sediments within the reservoir included residual Preclassic soil overtopped by Late Classic to Terminal Classic sediments and later materials, a clear indication that the reservoir tank was periodically dredged or flushed during the Early and Late Classic periods. Well-preserved varved sediments uncovered beneath dam wall collapse in the Palace Reservoir exhibit thin bands of organic sediment, probably derived from leaves falling into the reservoir in the dry season interbedded with lenses of carbonate sediment probably derived from the weathering of plastered surfaces, representing successive dry seasons (20). Many Neotropical plants in this region are deciduous because of the dramatic seasonal fluctuations in water availability. While it is possible that some leaves may have blown into the reservoir from vegetated land lying south and downslope from the South Acropolis and Temple V, we believe it is more likely that these leaves originated from plants in the immediate vicinity of the reservoir system, especially the ridge of unbuilt land between the Palace and the Temple Reservoir and Spring Pool, as well as the south flank of the Palace Reservoir.

Excavation operations 6O, 6L and 6K in the Palace Reservoir were formed by a series of three pits linked together into a trench (Fig. 2). This trench was located some 10 m west of the present-day interior dam of the Palace Reservoir and ran south from the current topographic low onto a gradual rise in the reservoir floor. Four distinct features are visible in the excavation profile. First, at the lowest part

of the trench near its north end is a narrow channel incised into bedrock and filled with a very dark organic silty clay, likely an aquic soil formed with a pooling point in the ravine channel (20). Stratigraphically above this basal clay is a zone of light gray silty clay formed by sediment deposited in the stagnant pool behind the reservoir dam; ceramic sherds embedded within this clayey sediment indicate that it was deposited during the Terminal Classic period (850-900 CE) grading upward into the Early Postclassic (900–1100 CE). Interfingering with the clayey reservoir pool sediments are a series of coarse sediments (mainly sand and gravel sized particles) in the form of thin debris flows that entered the reservoir from the south. These flows likely originated from collapses along the very steep southern embankment of the reservoir. Several larger debris flows are visible on the lidar-derived image of the Palace Reservoir (Fig. 1). Charcoal embedded within one of the lower debris flows produced a calibrated radiocarbon date range of 610-680 CE consistent with Late Classic ceramic sherds recovered from multiple flows. The material in the flows can be considered intrusive older construction fill originating in the decaying south wall of the reservoir. Notably, a section of the interior wall of the Palace dam is also known to have collapsed into the reservoir pool then subject to a buttressing repair (12, 20). The uppermost stratum revealed in the trench is the modern soil formed during the Postclassic period and later sediment and organic matter, reflecting a period at which time the reservoir was no longer effectively impounding water.

SI Materials and Methods

Because eDNA is typically highly degraded in archaeological contexts (21), obtaining long sequence reads generally is not possible. The 18S rRNA gene has been used elsewhere to study eukaryotic biodiversity (22). Highly conserved flanking regions allow for development of universal primers to amplify the informative variable sequences in rRNA. An added advantage is that ribosomal sequences are repeated in the genome, which increases the probability of amplification. Differences in 18S rRNA genes, however, have not proved to be sufficient to resolve plant taxa. In our previous studies (17), fungal species were abundant in reservoir sediments. Fungi can metabolize decaying matter and can proliferate in the sediments of the reservoirs, increasing their biomass compared to the plants. Extremely deep sequencing would have been needed to detect the rare plant genes under these circumstances. In this study, we primarily targeted plastid genes, unique to plants, from multiple regions and protein coding genes (rbcL, rp110, ycf1 and matK), non-coding spacers (trnT-trnL, trnL-trnF, and trnH-psbA), and the internal transcribed spacer regions (nrITS) of nuclear ribosomal DNA. Similar sets of target genetic markers have been successful in previous ancient vegetation studies (23-25).

Sediment samples from stratigraphic layers within four Tikal reservoirs and several *aguadas* (ponds) were collected during excavations in 2010. To avoid contamination from modern soils, and eliminate cross contamination from other strata, we collected samples in a column (26) from a selected characteristic

stratigraphic profile in each pit or excavation unit. This was accomplished by first shaving each profile with a sharp, clean trowel where the samples would be collected. Samples were taken in 10 cm increments starting at the bottom of the pit and working toward the top. Sediment samples destined for molecular and pollen analyses were extracted from the freshly shaved wall surface, placed in sterile plastic bags (Whirl-Packs) and labeled. In addition, flotation samples (2 liters each) were collected from the same profile after we finished collecting the pollen and molecular samples. This approach gave us the benefit of micro-remain and macro-remain plant data from each context. The macro-remain samples were processed by water flotation (27) and the pollen samples were sent to a laboratory in the US for pollen extraction. At the pollen lab, 2 g of sediment were removed from each sample in a laminar flow hood with a sterile spatula then the bags were immediately resealed. Results of the pollen and macrobotanical analyses have been published previously (27-29). Although we did have success extracting and identifying significant amounts of pollen from the ponds (*aguadas*) in the seasonal swamps (*bajos*) near Tikal, we were unsuccessful at doing so from the site core reservoir samples.

After arrival at the University of Cincinnati Paleoethnobotanical Laboratory, 30 samples designated for molecular genetic analysis were placed in a -80° C freezer until processing could begin. Just prior to DNA extraction, samples were thawed to 4° C then inserted, under sterile conditions, into tubes with glass beads then sealed prior to homogenization in a bead-beating machine.

DNA was extracted from sediment samples using DNeasy PowerSoil Kits (Qiagen) as described previously (17, 30-31). In brief, archaeological samples were extracted in a DNA clean lab facility dedicated to the study of cyanobacteria at the University of Cincinnati Department of Biological Sciences. Because of the general focus of this lab on cyanobacteria, it was highly unlikely that our samples would be contaminated with plant DNA from other experimental efforts. Nevertheless, rigorous protocols to avoid contamination were employed, including standardized workflow procedures, the wearing of personal protective equipment, and the preparation and processing of negative controls. Specimens underwent DNA extraction in batches of four each. The Uaxactun garden sample was extracted separately from the other samples discussed in this study and the hood was sterilized with bleach and 70% ethanol between each extraction. As always, established lab protocols (32) designed to avoid cross-contamination were followed scrupulously. This method was employed to successfully extract DNA from Tikal archaeological samples. The DNA content of samples was initially evaluated by Qubit Fluorometer Quantitation.

In order to process the fragmented genetic data recovered from the DNA extractions, we employed the services of RAPiD Genomics LLC (Gainesville, FL). Upon arrival of our 29 archaeologial extracts at the RAPiD Genomics facility, our samples were analyzed, under sterile conditions, using PicoGreen technology (Molecular Probes, Eugene OR), a commonly used assay for

fluorescence enhancement coupled with spectrophotometry for accurate dsDNA quantitation (33). Control DNAs used in this process were from commercial sources. In the spectrophotometer each sample was read against a blank 100 µl quartz microcuvette containing only TE buffer. From these assays we learned that 8 archaeological samples and our control sample from the Uaxactun garden contained sufficient DNA for whole genome amplification and library preparation. The results of this assay can be found in Table S10.

In addition, RAPiD Genomics was enlisted to design genetic probes that could capture common plant genes across a variety of taxa. To help create the probes, we provided sequences from GenBank for the regions *nrITS*, *matK, ndhF, psbA-trnH, rbcL, rpl10, trnL-trnF, trnT-trnL,* and *ycf1* for 68 species that are known, or suspected, to have been used by the ancient Maya (see Tables S1 and S9). These regions were selected because they have been well documented for variability at the genus and/or species level, and because they are well represented in GenBank. From these sequences, RAPiD Genomics designed probesets that they used to enrich DNA from our sediment extractions using their Capture Seq protocol prior to high-throughput Illumina sequencing. Following are the details of the probe design, the library preparation and the bioinformatics analysis performed.

The probes, which focused on plastid and nuclear genes, were based on the sequences we provided and were arranged in a 3x tiling array. All possible probes were designed in silico on the candidate genes (common plant genes across a variety of taxa), with start-end coordinates provided as target subsequences. From the total of all possible probes within the candidate sequences, no filters were applied to select a set of 11,391 probes that were used for hybridization. Briefly, DNA was sheared initially to a mean fragment length of 400 bp; fragments were end-repaired, followed by incorporation of unique dual-indexed Illumina adapters and PCR enrichment. Sequence capture and library preparation were performed utilizing their high-throughput workflow with proprietary chemistry. Following the probe capture process, only 6 of our 8 remaining archaeological specimens and the control sample were shown to contain adequate eukaryotic DNA to merit sequencing. Of the 6 archaeological samples with eukaryotic DNA, 4 were chosen because of their high archaeological significance (the other 2 were from A horizons) leaving us with five samples for sequencing including the Uaxactun control sample.

Regarding negative controls, these were carried out through the full sample processing pipeline alongside commercial samples as positive controls. These controls were evaluated for potential contamination at routine QC checks, which included DNA quantification and other proprietary measures. In general controls, both positive and negative, were included in library preparation to ensure successful library construction, these control samples typically are evaluated following library preparation and are typically not sequenced. No evidence of

contamination or other challenges were observed via the positive or negative controls for this project.

Parenthetically, the five remaining samples provide some indication about the possible effects of degradation of DNA. The garden sample had 251 identifiable DNA sequences while the four archaeological samples had an average of 32.5 sequences per sample so obviously there were degradation factors at work, but fortunately there was still enough DNA preserved in the archaeological sediments to generate useful data.

The five remaining samples with adequate amounts of eukaryotic DNA and high archaeological interest were pooled equimolar and sequenced using HiSeq 2x150. Raw sequencing reads were trimmed using Trimmomatic software to remove any sequencing adapters. (http://www.usadellab.org/cms/uploads/supplementary/Trimmomatic/TrimmomaticManual_V0.32.pdf)

Each sequencing run was performed in triplicate and each run included positive and negative controls resulting in ~500 total runs. Following quality control, merging of contigs, and removal of chimeras and tiny fragments, we recovered DNA sequences with an average length of 253 bp. At a sequence identity of 97%, we recovered 380 Operational Taxonomic Units (OTUs). The taxa-specific gene abundance determined from these OTUs aided in discerning the vascular plant vegetation surrounding both reservoirs.

A sliding window with a minimum quality score of 30 and a minimum accepted length of 50 bp were the parameters used. Trimmed reads were aligned to the target sequences with the Burrows-Wheeler alignment algorithm (34) using default settings. Reads that mapped to the target sequences were then assembled using SPAdes http://cab.spbu.ru/files/release3.12.0/manual.html). The resulting assemblies from SPAdes were presented to the authors for the identification of sequences (nodes).

To identify the plant DNA sequences generated by Rapid Genomics, we used the Basic Local Alignment Search Tool (BLAST) algorithm and the GenBank nucleotide database of the National Center for Biotechnology Information (NCBI). BLAST hits were initially filtered by Bitscore and Evalue to include only the best hits for each of the assembled sequences for every sample. BLAST hits were further filtered (35,36) to eliminate hits involving plants that were not native or likely domesticates (37) in Precolumbian Guatemala. To make systematic use of the BLAST algorithm, we closely followed the decision tree presented in Fig. S1 to interpret results consistently and conservatively.

Fig. S1. Decision tree for interpreting National Center for Biotechnology Information (NCBI) BLAST searches. Our eDNA gene sequences were compared to the NCBI database and their BLAST program evaluated the closest fit for our gene sequences by generating a Bitscore and Evalue. Generally, the higher the Bitscore the closer the match of our unknown sequences to sequences from plant species stored in the database. In numerous cases, only one plant species would be listed with the highest Bitscore. If the plant identified was native to Guatemala (35,36) or was a known New World cultigen (37), then our identification was clear and definitive. Because gene sequences can be conserved among even distantly related plants, however, it was not uncommon to find more than one species with a top ranked Bitscore. When this occurred, we followed the decision tree outlined below. Note that in all cases, we took a conservative approach and assigned a broader taxon if there was any ambiguity.

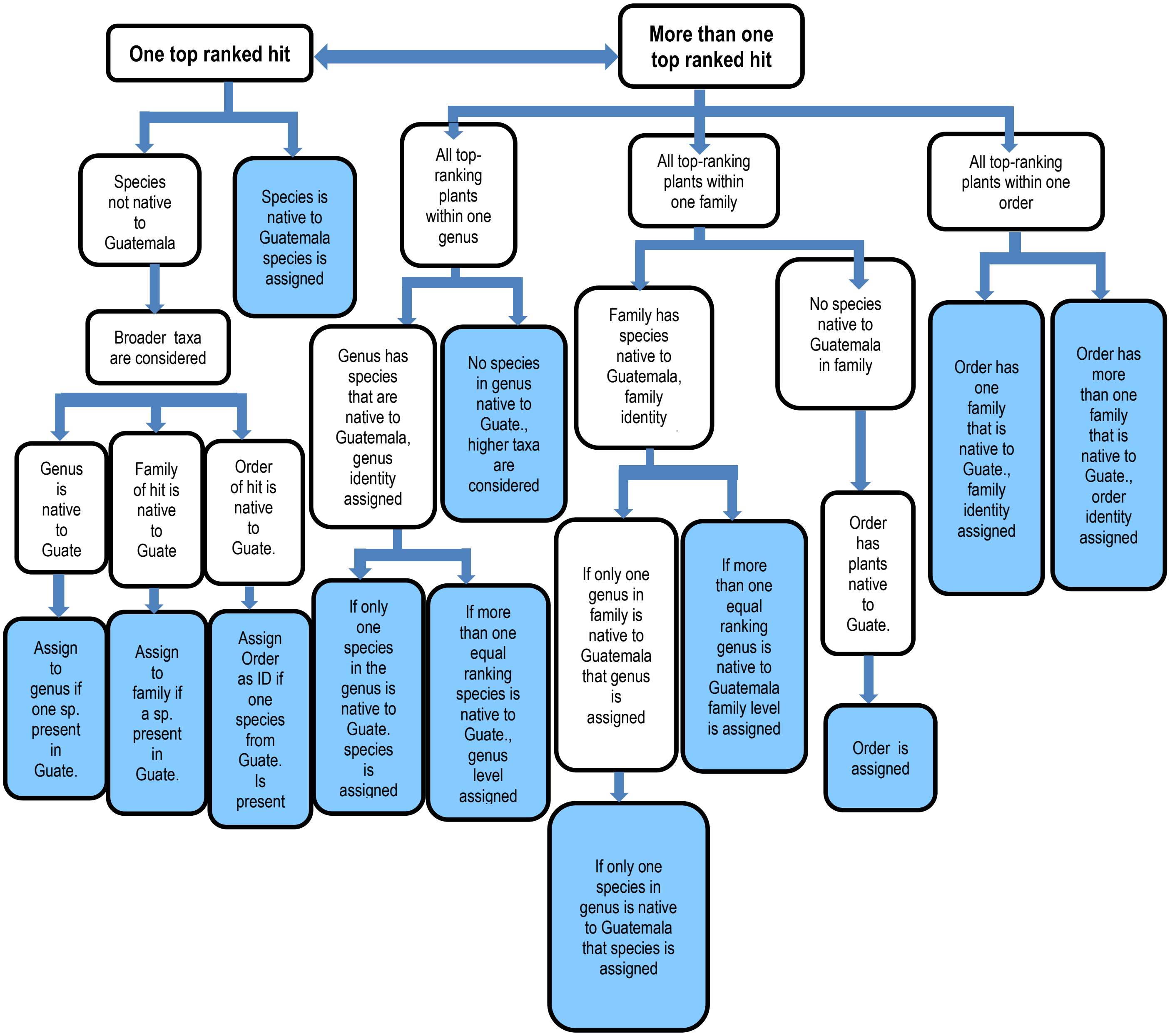

Table S1. Plants identified from ancient Tikal using pollen and macro-remain analyses (27,28)**.** Chronological assessments (C) are abbreviated as follows: 1=Middle Preclassic (1000-300 BCE), 2=Late Preclassic (300 BCE-250 CE), 3=Early Classic (250-600 CE), 4=Late Classic (600-850 CE), 5=Terminal Classic (850-900 CE), 6=Postclassic (900-1150 CE), 7=unknown date. H=habitat where the plant species likely originated: 1=upland forest, 2=bajo, 3=kitchen garden, 4=field, 5=other; N=number of contexts from which the plant remains were recovered; P=plant part: 1=wood, 2=seed, 3=pit, 4=endocarp, 5=tuber, 6=stem, 7=cob, 8=pollen, 9=leaf; † see (38), ‡ see (39).

Taxon	Common Name	Contexts
Anacardiaceae		
Metopium brownei (Jacq.) Urb	black poison wood	C4;H2;N1;P1
Astronium graveolens Jacq.	glassy wood	C2-4;H1;N1;P1
Spondias cf. *purpurea* L.	jocote	C2-5;H1,3;N8;P1,3
Tapirira mexicana Marchand	tanto	C2-4;H1;N2;P1
Apocynaceae		
Aspidosperma spp.	white malady	C2-4;H1;N6;P1
Cameraria latifolia L.	white poison wood	C2;H2;N1;P1
cf. *Lacmellea* sp.	chicle dwarf	C7;H2;N1;P1
Tabernaemontana sp.	cojotón	C2-4;H1;N1;P1
Tecoma stans (L.) H.B.K.	flor amarilla	C3,4;H3,5;N1;P1
Thevetia ahouai (L.) A. DC.	cocheton	C2;H1;N1;P2
Araceae		
Xanthosoma sagittifolium(L.)Schott.	malanga	C4,5;H3,4;N1;P5‡
Arecaceae		
Acrocomia aculeata Lodd. ex Mart.	coyol	C2-4;H3;N1;P4
Bactris major Jacq.	biscoyol	C3;H2,3;N2;P3,6
Bignoniaceae		
cf. *Tabebuia* sp.	yellow mayflower	C4;H1;N2;P1
Burseraceae		
Protium copal (Schl. & Cham.) Engl.	copal	C2-4;H1;N6;P1,2
Cannaceae		
Canna cf. *indica* L.	achira	C2;H2;N1;P8
Chrysobalanaceae		
Hirtella sp.	pigeon plum	C2-4;H1;N1;P1
Clusiaceae		
Clusia sp.	matapalo	C2;H1;N1;P1
Garcinia intermedia (Pittier) Hamm.	jocomico	C5;H5;N1;P1
Combretaceae		
Terminalia buceras (L.) C. Wright	pukté	C3;H2;N3;P1
Convolvulaceae		
Ipomoea batatas (L.) Lam.	sweet potato	C1;H3,4;N1;P5
Cucurbitaceae		
Cucurbita moschata (Lam.) Poir.	squash	C4;H3-4;N1;P2†
Cucurbita pepo L.	pumpkin	C3;H3-4;N3;P2
Cyperaceae		
Cyperus canus J. Presl & C. Presl.	tule	C4;H2;N1;P9

Table S1. Continued

Taxon	Common Name	Contexts
Euphorbiaceae		
Croton sp.	hierba de jabali	C2,3;H2;N3;P1
Sebastiania sp.	white poison wood	C2-4;H1;N3;P1
Fabaceae		
Acacia sp.	subín	C7;H1;N1;P1
Acosmium panamense (Benth.)Yak.	billy webb	C3;H1;N2;P1
Caesalpinia sp.	warree wood	C4;H1;N3;P1
Enterolobium cyclocarpum(Jacq.)Gr.	guanacaste	C5;H1;N1;P2†
Erythrina spp.	tiger wood	C3,4;H3,5;N2;P1
Gliricidia sepium (Jacq.) Steud.	madre de cacao	C4;H2;N2;P1
Haematoxylum campechianum L.	logwood	C3-6;H2;N31;P1
Phaseolus coccineus L.	scarlet runner bean	C2-3;H3-4;N2;P2
Phaseolus lunatus L.	lima bean	C3;H3-4;N2;P2
Phaseolus vulgaris L.	common bean	C3;H3-4;N2;P2
Piscidia piscipula (L.) Sarg.	habin	C4;H2,5;N1;P1
Lauraceae		
Licaria campechiana (Standl.) Kost.	laurelillo	C2-4;H1-2;N8;P1
Lonchocarpus spp.	dogwood	C2-6;H1,2;N5;P1
Nectandra spp.	timber sweet	C2-4;H1,2;N8;P1
Ocotea puberula (Rich.) Nees.	wakkowit	C2-4;H1;N2;P1
Persea americana Mill.	aguacate	C5;H3;N1;P3
Malpighiaceae		
Byrsonima crassifolia (L.) H.B.K.	nance	C2-6;H3;N2;P1,3
Malvaceae		
Ceiba pentandra (L.) Gaertn.	ceiba	C5;H1;N1;P1
Gossypium hirsutum L.	algodon	C3;H3;N1;P2
Heliocarpus sp.	broadleaf moho	C2,3;H1;N2;P1
Theobroma cacao L.	cacao	C3-5;H3;N4;P1,2
Meliaceae		
cf. *Carapa guianensis* Aubl.	andiroba	C2,3;H1;N1;P1
Guarea glabra Vahl.	cedrillo	C2-4;H1;N1;P1
Trichilia hirta L.	red cedar	C3;H1;N3;P1
Moraceae		
Brosimum alicastrum Sw.	breadnut, ramón	C2-5;H1;N8;P1,2
Ficus sp.	fig	C3-4;H1;N2;P1
Pseudolmedia glabrata(Lieb.) Berg	cherry	C2-4;H1;N8;P1
Trophis sp.	white ramón	C3-5;H1;N4;P1
Myrtaceae		
Eugenia spp.	guabillo	C2-4;H1;N3;P1
Pimenta dioica (L.) Merr.	allspice	C5;H1;N1;P2
Picramniaceae		
Alvaradoa subovata Cronquist	cortacuero	C4,5;H5;N2;P1
Pinaceae		
Pinus spp.	pino,pine	C2-5;H5;N118;P1
Piperaceae		
Piper sp.	cordoncillo	C3;H1;N2;P1

Table S1. Continued

Taxon	Common Name	Contexts
Poaceae		
Zea mays L.	maize	C3-6;H3-4;N33;P2,6,7,8
Rubiaceae		
cf. *Guettarda combsii* Urb.	arepa	C7;H1;N2;P1
cf. *Morinda* sp.	pinuela	C3;H3;N1;P1
Psychotria sp.	night bloom	C4;H1;N1;P1
Rutaceae		
Zanthoxylum caribaeum Lam.	prickly yellow	C2-4;H1,2;N4;P1
Salicaceae		
Casearia laetioides (A.Rich.)Warb.	drunkan bayman wood	C2-;H1;N1;P1
Casearia sp.	café de monte	C2;H1;N1;P1
Salix cf. *chilensis* Molina	willow	C5;H2;N2;P1
Sapindaceae		
Cupania sp.	grande betty	C3-5;H1,2;N1;P1
Sapotaceae		
Chrysophyllum sp.	caimito	C2-4;H1;N2;P1
Pouteria sapota (Jacq.)Moore.& Strn.	sapote	C4;H1,3;N1;P1
Pouteria spp.	mamey	C2-5;H1;N20;P1
Manilkara zapota (L.) P. Royen	sapodilla	C2-5;H1-3,N34,P1,2
Sideroxylon sp.	silion	C2-4;H1;N6;P1
Ulmaceae		
Ampelocera hottlei (Standl.) Standl.	bullhoof	C2-4;H1;N1;P1
Celtis iguanaea (Jacq.) Sarg.	sits-muk	C2-5;H1;N1;P3†

Table S2. Background data generated by the National Center for Biotechnology Information for the results reported for WA07 (Early Preclassic, Palace Reservoir).

Family/Order	Assigned taxon	GenBank Accession#	Node	Length	Bitscore	Evalue	%ID
Amaryllidaceae	Allium sp.	JF972893.1	37746	260	459	2.38E-125	98.46%
Apocynaceae	Apocynaceae	MG963247.1	745	650	865	0	96.56%
Apocynaceae	Apocynaceae	DQ522595.1	5115	392	675	0	97.70%
Apocynaceae	Apocynaceae	AF130168.1	45781	253	451	3.86E-123	98.81%
Apocynaceae	Apocynaceae	KJ485850.1	539	691	893	0	99.59%
Apocynaceae	Apocynaceae	KP208925.1	19175	280	427	7.30E-116	97.59%
Apocynaceae	Apocynaceae	MG963264.1	203	836	1229	0	95.15%
Apocynaceae	Apocynaceae	MG963248.1	15637	286	477	1.00E-130	96.84%
Apocynaceae	Cascabela thevetia	AF130169.1	8263	341	571	8.00E-159	97.05%
Apocynaceae	Tabernaemontana donnell-smithii	GU973907.1	64279	238	440	2.00E-119	100%
Arecaceae	Arecaceae	KT312929.1	11332	308	534	4.66E-148	98.05%
Asparagaceae	Dracaena sp.	NC_039776.1	4916	397	464	2.00E-126	95.85%
Asteraceae	Asteraceae	AY215778.1	80500	226	407	7.43E-110	100%
Asteraceae	Asteraceae	AP007232.1	3975	422	523	9.16E-145	100%
Meliaceae	Cedrela odorata L.	NC_037251.1	89853	219	399	2.00E-107	99.54%
Moraceae	Brosimum alicastrum	JQ588387.1	3809	427	761	0	98.83%
Moraceae	Brosimum alicastrum	AF501601.1	12719	296	523	2.00E-144	98.64%
Moraceae	Ficus aurea	KJ773509.1	6004	375	671	0	98.93%
Moraceae	Ficus insipida	GQ504531.1	34539	263	486	2.00E-133	100%
Moraceae	Ficus sp.	KY635880.1	77815	228	422	5.00E-114	100%
Moraceae	Ficus sp.	KP752397.1	43427	255	460	1.00E-125	99.22%
Moraceae	Ficus sp.	KY416513.1	2862	461	743	0	99.51%
Moraceae	Ficus sp.	MT093220.1	11895	303	544	1.00E-150	99.01%
Moraceae	Moraceae	KU355297.1	476	709	1225	0	97.88%
Moraceae	Moraceae	AF500346.1	85912	222	385	7.00E-103	99.07%
Moraceae	Moraceae	NC_047236.1	425	725	1206	0	96.69%
Moraceae	Trophis sp.	MH135781.1	7	1471	1467	0%	88.57%
Poaceae	Stipa sp.	KY826234.1	57812	243	420	2.00E-113	97.94%
Zingerberales	Zingerberales	HF677508.1	5712	380	568	4.57E-158	100%

Table S3. Background data generated by the National Center for Biotechnology Information for the results reported for WA09 (Late Classic, Temple Reservoir).

Family/Order	Taxon	GenBank Accession #	Node	Length	Bitscore	E value	%ID
Amarylidaceae	Allium sp.	KF728079.1	96221	222	411	5.63E-111	100.00%
Apocynaceae	Apocynaceae	AJ419739.1	185	818	859	0.00E+00	99.58%
Apocynaceae	Apocynaceae	KJ953907.1	1	4617	1925	0	98.02%
Apocynaceae	Apocynaceae	HQ634605.1	9	1508	1535	0	99.30%
Apocynaceae	Apocynaceae	KJ953913.1	218	789	508	2.67E-140	97.96%
Apocynaceae	*Lacmellea* sp.	MG963264.1	11	1455	2381	0	98.31%
Apocynaceae	*Rauvolfia* sp.	NC_047244.1	2	4075	6499	0	95.59%
Arecaceae	Arecaceae	KT312918.1	79663	233	431	4.57E-117	100.00%
Arecaceae	*Cryosophila stauracantha*	KY020641.1	31440	268	484	8.00E-133	99.25%
Asclepiadaceae	*Cynanchum* sp.	MH410146.1	7	1597	2039	0	97.42%
Asteraceae	*Bidens alba*	MF159499.1	22274	276	407	9.36E-110	100.00%
Cannabaceae	*Trema* sp.	D86313.1	12035	296	525	5.00E-145	98.65%
Fabaceae	*Lonchocarpus* sp.	KX816373.1	111297	213	377	1.00E-100	98.59%
Moraceae	*Brosimum alicastrum*	AY289328.1	35037	264	446	4.00E-121	97.33%
Moraceae	*Brosimum* sp.	FJ039163.1	115945	210	387	8.88E-104	100.00%
Moraceae	*Ficus* sp.	EU091565.1	55	974	1249	0	97.05%
Moraceae	*Ficus* sp.	MN364706.1	592	651	1186	0	99.54%
Poaceae	Poaceae	KU291482.1	50402	252	466	1.37E-127	100.00%
Zingiberales	Zingiberales	KY753133.1	61137	245	453	1.03E-123	100.00%

Table S4. Background data generated by the National Center for Biotechnology Information for the results reported for WA01 (Terminal Classic, Palace Reservoir).

Family/Order	Taxon	GenBank Accession#	Node	Length	Bitscore	E-value	%ID
Actinidaceae	Saurauia sp.	NC_044098.1	8420	282	472	7.00E-129	96.81%
Apocynaceae	Apocynaceae	KX364402.1	5119	300	532	1.63E-147	98.67%
Apocynaceae	Apocynaceae	AJ419739.1	304	616	861	0	99.37%
Apocynaceae	Apocynaceae	KR231887.1	188	685	544	7.74E-151	99.33%
Apocynaceae	Tabernaemontana sp.	HQ634605.1	2544	364	621	8.00E-174	99.42%
Arecaceae	Arecaceae	KT312915.1	51118	224	409	2.05E-110	99.55%
Arecaceae	Cryosophila sp.	AJ404747.1	56021	219	394	1.00E-105	99.09%
Boraginaceae	Boraginaceae	GQ997232.1	63445	211	361	5.35E-96	97.63%
Boraginaceae	Boraginaceae	EU599824.1	5060	301	329	2.26E-86	100%
Gentianales	Gentianales	EU650387.1	1958	388	303	1.62E-78	97.21%
Gentianales	Gentianales	KJ485850.1	121	758	881	0	99.18%
Meliaceae	Meliaceae	KF840441.1	1519	414	459	8.00E-125	99.60%
Moraceae	Ficus sp.	EU091565.1	185	691	800	0	99.10%
Moraceae	Ficus tonduzii	AY730140.1	2998	350	638	8.00E-179	99.43%
Moraceae	Moraceae	KU355297.1	65455	209	387	8.83E-104	100%
Moraceae	Morus celtidifolia	NC_047236.1	61339	213	394	1.00E-105	100%
Moraceae	Trophis racemosa	HM747178.1	4723	307	562	4.00E-156	99.67%
Moraceae	Trophis racemosa	HM747178.1	5535	293	529	4.00E-146	100%
Poaceae	Imperata sp.	KU291466.1	65456	209	387	2.00E-103	100%
Poaceae	Poaceae	KU961860.1	5001	302	558	2.70E-155	100%
Poaceae	Poaceae	KU961859.1	43022	232	429	1.64E-116	100%
Poaceae	Poaceae	KF184927.1	2189	377	669	0	98.68%
Poaceae	Poaceae	KU291447.1	24172	255	472	2.99E-129	100%
Zingiberales	Zingiberales	HF677508.1	34971	242	448	4.74E-122	100%
Zingiberales	Zingiberales	MH603425.1	52239	223	379	3.00E-101	97.31%

Table S5. Background data generated by the National Center for Biotechnology Information for the results reported for WA08 (Early Post Classic, Palace Reservoir).

Family/Order	Taxon	GenBank Accession #	Node	Length	Bitscore	E-value	%ID
Amaryllidaceae	Allium sp.	KU318712.1	32832	257	396	1.53E-106	99.54%
Anacardiaceae	Spondias sp.	KU756561.1	80697	216	399	1.18E-107	100%
Apocynaceae	Apocynaceae	AJ419739.1	22	1238	1886	0	99.14%
Apocynaceae	Apocynaceae	Z98187.1	11332	287	497	5.64E-137	97.91%
Apocynaceae	Apocynaceae	MG963234.1	431	659	1092	0	96.66%
Apocynaceae	Apocynaceae	MG963234.1	15	1516	2185	0	95.83%
Apocynaceae	Cascabela thevetia	AF130169.1	204	769	1376	0	98.96%
Apocynaceae	Cascabela thevetia	AF130169.1	3374	401	719	0	99.00%
Apocynaceae	Cascabela thevetia	AF130169.1	6046	342	436	1.07E-118	98.01%
Apocynaceae	Cynanchum sp.	MH410146.1	45	992	1526	0	97.96%
Apocynaceae	Tabernaemontana sp.	GU973907.1	3623	394	728	0	100%
Arecaceae	Arecaceae	KT312927.1	37947	252	416	1.40E-112	97.54%
Arecaceae	Arecaceae	KT312922.1	47640	243	449	1.33E-122	100%
Arecaceae	Arecaceae	KT312929.1	3876	387	715	0	100%
Arecaceae	Arecaceae	KX640894.1	6861	329	608	2.90E-170	100%
Arecaceae	Cryosophila stauracantha	HQ720625.1	9408	296	542	5.00E-150	99.66%
Arecaceae	Sabal mauritiiformis	KY020654.1	10354	289	534	8.00E-148	100.00%
Arecaceae	Sabal sp.	KF928963.1	59239	233	431	4.57E-117	100%
Arecaceae	Sabal sp.	KF928963.1	49867	241	446	1.70E-121	100%
Arecaceae	Sabal sp.	KF928963.1	91115	208	385	6.00E-103	100%
Arecaceae	Sabal sp.	MG647087.1	92478	207	383	2.00E-102	100%
Bignoniaceae	Bignoniaceae	FJ887849.1	8530	306	527	7.74E-146	97.71%
Bignoniaceae	Bignoniaceae	KC914586.1	22255	269	494	6.78E-136	100%
Bignoniaceae	Bignoniaceae	KX636161.1	799	583	634	8.06E-178	97.08%
Bignoniaceae	Bignoniaceae	KP863525.1	8285	309	355	3.82E-94	97.60%
Bignoniaceae	Adenocalymma sp.	MG749191.1	85888	212	392	4.00E-105	100%
Bignoniaceae	Amphilophium paniculatum	NC_042918.1	889	565	1011	0	98.94%
Bignoniaceae	Amphilophium paniculatum	NC_042918.1	31903	258	472	6.00E-129	99.61%
Bignoniaceae	Amphilophium sp.	NC_042934.1	2900	419	758	0	99.28%
Cannabaceae	Celtis iguanaea	MN381778.1	10788	288	501	9.00E-138	98.93%
Cannabaceae	Celtis iguanaea	MN381778.1	5877	345	553	3.00E-153	100%
Cucurbitaceae	Cucurbitaceae	KX231331.1	56949	235	424	7.73E-115	99.14%
Moraceae	Moraceae	HG963667.1	31	1101	782	0	100%
Moraceae	Moraceae	AF501601.1	24	1217	1772	0	100%
Moraceae	Moraceae	AY289329.1	4	2976	3603	0	98.96%
Moraceae	Moraceae	NC_047182.1	138	811	396	1.00E-105	99.54%

Table S5. Continued

Family/Order	Taxon	Accession #	Node	Length	Bitscore	E-value	%ID
Moraceae	Brosimum alicastrum	AF500346.1	3184	408	726	0	98.55%
Moraceae	Brosimum guianense	AY635481.1	6268	338	551	1.00E-152	97.52%
Moraceae	Brosimum guianense	FJ037845.1	2228	450	728	0	95.79%
Moraceae	Morus celtidifolia	NC_047236.1	2180	453	793	0	98.23%
Poaceae	Poaceae	KU961859.1	91114	208	383	1.13E-102	100%
Poaceae	Poaceae	KX507245.1	84554	213	394	5.40E-106	100.00%
Rosales	Rosales	AF479098.1	3875	387	272	1.76E-69	98.68%
Rosales	Rosales	KX527244.1	43	1009	553	3.64E-153	97.546
Rubiaceae	Psychotria sp.	FJ208594.1	67883	226	370	2.00E-98	95.13%
Sapindaceae	Sapindaceae	AB586576.1	33831	256	462	1.81E-126	99.22%
Sapindaceae	Sapindaceae	KY635881.1	76837	219	405	2.58E-109	100%
Sapotaceae	Pouteria sp.	KJ399421.1	4488	372	654	0	98.39%
Zingerberales	Zingerberales	HF677508.1	154	798	1177	0	99.84%
Zingerberales	Zingerberales	HF677508.1	857	572	959	0	100%
Zingerberales	Zingerberales	HF677508.1	6	2369	2230	0	99.92%
Zingerberales	Zingerberales	HF677508.1	8	2022	2848	0	100%
Zingerberales	Zingerberales	HF677508.1	26	1188	1629	0	99.75%
Zingerberales	Zingerberales	HF677508.1	3	2986	3524	0	100%
Zingerberales	Zingerberales	HF677508.1	5	2644	3884	0	100%
Zingerberales	Zingerberales	HF677508.1	14	1532	2346	0	99.84%
Zingerberales	Zingerberales	LT576835.1	1893	471		7.20E-110	100%

Table S6. Accelerator mass spectrometry (AMS) radiocarbon (^{14}C) dates for Tikal reservoirs and related contexts discussed in this text. AMS dates were obtained from Beta Analytic (Miami, FL, USA) and the National Ocean Sciences Accelerator Mass Spectrometry Facility (Woods Hole, MA, USA). Data provided include AMS radiocarbon sample composition (SOM = soil organic matter), provenience, depth in cm, measured radiocarbon years before present (BP), and calibrated age at two sigma margins for error. Samples were collected from wet and dry cores and excavation profiles. These data were extracted from larger tables published previously (17,40).

Lab Number	Composition	Provenience	Depth (cm)	Measured ^{14}C (yr BP)	Calibrated Age (2 σ)
Beta-281750[a]	Charcoal	Palace (Op 6Q)	Above dam collapse	1,250 ± 40	670-880 CE
Beta-281751[a]	Charcoal	Palace (Op 6Q)	Below dam collapse	1,260 ± 40	660-880 CE
Beta-288914[a]	Charcoal	Palace (Op 6L)	150	1,380 ± 40	610-680 CE
Beta-281749[a]	Charcoal	Palace (Op 6U)	Dam fill	15,360±50	16860-16740BCE
Beta-281745[a]	SOM	Palace (Op 6O)	Channel fill	3,410 ± 40	1780-1620 BCE
88638[a]	SOM	Palace (Op 6J-13 Core 1-1)	50-60	3,460 ± 30	1739-1535 BCE
88682[a]	SOM	Palace (Op 6J-13 Core-2)	100-110	2,150 ± 40	358-55 BCE
Beta-281746[a]	Charcoal	Temple Main Tank (Op 7C)	110	1,200 ± 40	680-890 CE

Table S6. Continued.

Lab Number	Composition	Provenience	Depth (cm)	Measured ^{14}C (yr BP)	Calibrated Age (2 σ)
85584[a]	SOM	Temple Main Tank (Op 7C)	130-140	1,230 ± 25	721-839 CE
85585[a]	SOM	Temple Main Tank (Op 7C)	140-162	1,830 ± 25	143-215 CE
85583[a]	SOM	Temple Main Tank (Op 7C)	162-194	1,250 ± 35	701-811 CE
88676[b]	SOM	Temple Silting Tank (Op 7Core 23-2)	70-80	195 ± 35	1645-1952 CE
88677[b]	SOM	Temple Silting Tank (Op 7 Core 23-2)	110-120	2,330 ± 40	521-216 BCE
Beta-298985[a]	Charcoal	Temple Silting Tank (Op 7A)	130	1,370 ± 30	640-680 CE

a. Sample collected from an excavation profile.
b. Dry core with some compression.

Table S7. Plants physically observed in the Uaxactun household garden. Plants in bold represent species also identified from the DNA analysis.

Family	Genus/Species name	Common Name
Annonaceae	***Annona reticulata* L.**	anona colorada
	***Annona* sp.**	anona
Apocynaceae	***Cascabela thevetia* (L.) Lippold**	chilendron
	***Plumeria obtusa* L.**	zopilote
	***Plumeria rubra* L.**	flor de Maya
	***Tabernaemontana donnell-smithii* Rose**	huevos de caballo
Arecaceae	*Chamadorea* sp.	xate embra
	***Cocos nuciferea* L.**	coconut
	Sabal mauritiiformis (H. Karst) Griseb. & H. Wendl.	guano
Asparagaceae	*Dracaena trifasciata* (Prain) Mabb.	sanseveria
	***Yucca gigantea* Lem.**	isote
Asteraceae	*Cosmos caudatus* Kunth	flor de petenera
	***Pluchea* sp.**	siguapate
	***Tagetes erecta* L.**	xpuhuc
	Verbesina sp.	toquillo
Balsaminaceae	*Impatiens balsamina* L.	impatiens
Bignoniaceae	*Parmientiera aculeata* (HBK) Seem.	cuajilote
Boraginaceae	***Cordia sebestena* L.**	siracote
Burseraceae	***Bursera simaruba* (L.) Sarg.**	cha caa
Cactaceae	*Opuntia cochenillifera* (L.) Mill.	nopal
	Rhipsalis baccifera (J.S.Muell.) Stearn	pitaya
Caricaceae	*Carica papaya* L.	papaya
Casuarinaceae	*Casuarina equisetifolia* L.	si kin
Combretaceae	***Terminalia catappa* L.**	almendro
Costaceae	*Costus pulverulentus* C. Presl.	wild ginger
Crassulaceae	*Kalanchoe pinnata* (Lam.) Pers.	hojo de agua
Cucurbitaceae	*Sechium edule* (Jacq.) Sw.	chayote
Euphorbiaceae	*Croton* sp.	wild cimarron

Table S7. Continued.

Family	Genus/Species name	Common name
Fabaceae	***Delonix regia* (Bojer ex Hook.) Raf.**	guacamayo
	***Lonchocarpus guatemalensis* Benth.**	palo de gusano
	***Phaseolus vulgaris* L.**	frijoles, beans
	Piscidia piscipula (L.) Sarg.	jabin
Lauraceae	***Persea americana* Mill.**	aguacate
Malvaceae	***Guazuma ulmifolia* Lam.**	pi choy
Marantaceae	*Calathea lutea* (Aubl.) G. Mey.	hoja del sal
Meliaceae	*Trichilia* sp.	son
Musaceae	***Musa × paradisiaca* L.**	platano, banana
Piperaceae	***Piper aduncum* L.**	cordoncillo negro
Poaceae	***Zea mays* L.**	maize
Portulacaceae	*Portulaca oleracea* L.	purslane
Rubiaceae	***Hamelia patens* Jacq.**	ix kanan, coralillo
Rutaceae	*Citrus limon* (L.) Burm. F.	limon
	***Citrus aurantium* L.**	naranjo
Sapindaceae	*Blomia prisca* (Standl.) Lundell	tzol
Sapotaceae	***Manilkara zapota* (L.) P.Royen**	chico sapote
	***Pouteria sapota* (Jacq.) H.E.Moore & Stearn**	zapote
Simaroubaceae	*Simarouba glauca* DC.	aceituna
Urticaceae	*Cercropia* sp.	guarumo
Xanthorrhoeaceae	*Aloe vera* (L.) Burm. f.	savila

Table S8. Plants detected using the analysis of eDNA sequences recovered from a soil sample taken from a household garden in Uaxactun (sample WA05).

Family/ Order	Interpreted Taxon	Genbank Accession #	Node	Length	Bit score	Evalue	%ID
Acanthaceae	Pseuderanthemum sp.	KX526942.1	121981	233	425	2.13E-115	99.571
Acanthaceae	Ruellia sp.	AF531773.1	145032	224	403	9.52E-109	99.107
Amaranthaceae	Achyranthes	EF688734.1	37160	275	466	1.50E-127	97.455
Amaryllidaceae	Allium sp.	KT898250.1	196851	152	276	1.36E-70	99.342
Anacardiaceae	Spondias mombin L.	KY828469.1	132663	229	424	7.50E-115	100
Anacardiaceae	Spondias mombin L.	KY828469.1	41713	242	425	2.22E-115	98.347
Annonaceae	Annona sp.	KU563738.1	70407	255	464	4.98E-127	99.608
Annonaceae	Annona mucosa Jacq.	MT742547.1	76923	252	466	3.00E-127	100
Annonaceae	Annona mucosa Jacq.	MT742547.1	6	1917	7204	0	100
Annonaceae	Annona mucosa Jacq.	MT742547.1	571	738	1363	0	100
Annonaceae	Annona mucosa Jacq.	MT742547.1	1484	590	2192	0	100
Annonaceae	Annona mucosa Jacq.	MT742547.1	940	504	1221	0	100
Annonaceae	Annona mucosa Jacq.	MT742547.1	27103	280	518	8.00E-143	100
Annonaceae	Annona mucosa Jacq.	MT742547.1	122	975	1801	0	100
Annonaceae	Annona mucosa Jacq.	MT742547.1	1338	547	1120	0	99.84
Annonaceae	Annona mucosa Jacq.	KX663852.1	11647	321	588	3.74E-164	99.688
Annonaceae	Annona mucosa Jacq.	MT742547.1	3778	457	845	0	100
Annonaceae	Annona sp.	KU563738.1	49	832	1426	0	97.596
Annonaceae	Annona sp.	KU563738.1	28	1000	1807	0	99.3
Annonaceae	Annona sp.	KU563738.1	14015	308	569	1.28E-158	100
Annonaceae	Annona sp.	KU563738.1	3257	478	876	0	99.791
Annonaceae	Annona sp.	KU563738.1	16354	294	544	7.34E-151	100
Apocynaceae	Apocynaceae	KJ485850.1	5053	432	743	0	97.685
Apocynaceae	Aslepias sp.	KU041142.1	196026	192	344	4.87E-91	98.958
Apocynaceae	Cascabela thevetia L.	AF130169.1	35444	274	496	1.93E-136	99.27
Apocynaceae	Plumeria rubra L.	NC_046018.1	2976	492	832	0	97.154
Apocynaceae	Plumeria rubra L.	KX426215.1	17890	289	529	2.02E-146	99.654
Apocynaceae	Plumeria sp.	KX910893.1	14788	303	544	7.60E-151	99.01
Apocynaceae	Tabernaemontana donnell-smithii	GU973907.1	83	898	1629	0	99.443
Apocynaceae	Tabernaemontana sp.	DQ660640.1	196282	177	322	2.07E-84	99.435
Apocynaceae	Tabernaemontana sp.	KX426058.1	10629	336	603	1.38E-168	99.107
Arecaceae	Arecaceae	AJ404828.1	4516	433	800	0	100
Arecaceae	Arecaceae	KT312918.1	126953	231	427	5.85E-116	100
Arecaceae	Arecaeae	KT312918.1	12254	321	586	1.32E-163	99.688
Arecaceae	Cocos nucifera L.	KX028884.1	97253	243	449	3.00E-122	100

Table S8. Continued.

Family/ Order	Interpreted Taxon	Accession #	Node	Length	Bit score	Evalue	%ID
Arecaceae	Cocos nucifera L.	KX028884.1	60	308	558	9.67E-155	99.351
Arecaceae	Cocos nucifera L.	KX028884.1	7334	373	689	0	100
Arecaceae	Cocos nucifera L.	KX028885.1	72453	254	453	1.08E-123	98.819
Arecaceae	Cocos nucifera L.	KX028885.1	6516	356	636	1.58E-178	98.876
Arecaceae	Cocos nucifera L.	KX028884.1	4	2180	4013	0	99.908
Arecaceae	Cocos nucifera L.	KX028884.1	31151	277	499	1.51E-137	99.278
Arecaceae	Cocos nucifera L.	KX028884.1	296	779	1439	0	100
Arecaceae	Cocos nucifera L.	KX028885.1	261	862	1592	0	100
Arecaceae	Cocos nucifera L.	KX028885.1	196394	171	316	9.22E-83	100
Arecaceae	Cocos nucifera L.	KX028884.1	196176	183	333	9.95E-88	99.454
Arecaceae	Cocos nucifera L.	KX028884.1	145501	224	403	9.52E-109	99.107
Arecaceae	Cocos nucifera L.	KX028884.1	4222	442	817	0	100
Arecaceae	Cocos nucifera L.	KX028884.1	117691	235	429	1.66E-116	99.574
Arecaceae	Cocos nucifera L.	KX028884.1	11	1612	2961	0	99.814
Arecaceae	Arecaceae	AY044534.1	150905	173	320	7.23E-84	100
Asparagaceae	Asparagales	KX519714.1	137161	227	409	2.08E-110	99.119
Asparagaceae	Yucca gigantea Lem.	EU092488.1	152925	220	407	7.20E-110	100
Asparagaceae	Yucca sp.	MF963735.1	70461	255	438	6.00E-119	97.65
Asparagaceae	Yucca sp.	NC_045534.1	3283	477	843	0	98.532
Asteraceae	Asteraceae	JX113265.1	196720	158	289	1.82E-74	99.367
Asteraceae	Asteraceae	AY215797.1	6217	392	691	0	98.469
Asteraceae	Mikania micrantha Kunth	MG712604.1	116919	235	424	7.73E-115	99.149
Asteraceae	Parthenium hysterophorus L.	MT576959.1	4349	435	749	0	97.701
Asteraceae	Parthenium hysterophorus L.	MT576959.1	96703	202	374	8.24E-100	100
Asteraceae	Parthenium hysterophorus L.	MF135350.1	196211	181	322	2.11E-84	98.895
Asteraceae	Parthenium hysterophorus L.	AF384759.2	5167	370	673	0	99.459
Asteraceae	Parthenium hysterophorus L.	MT576959.1	4277	440	813	0	100
Asteraceae	Parthenium hysterophorus L.	AY215837.1	2608	510	931	0	99.608
Asteraceae	Pluchea carolinensis (Jacq.)Don	HG963499.1	62105	221	409	2.42E-110	100
Asteraceae	Tagetes erecta L.	MN203535.1	70478	166	291	8.90E-75	98.193
Bignoniacace	Crescentia cujete L.	KT182634.2	343	756	1295	0	97.751
Bignoniacace	Crescentia cujete L.	KT182634.2	15636	244	440	8.01E-120	99.18
Bignoniacace	Crescentia cujete L.	KT182634.2	151	833	1495	0	99.04
Bignoniaceae	Podranea ricasoliana (Tanfani)	MG831877.1	4809	222	398	5.17E-107	99.099
Boraginaceae	Boraginaceae	AF258345.1	196438	169	300	9.08E-78	98.817
Boraginaceae	Bourreria sp.	KF158048.1	14322	305	558	2.74E-155	99.672
Boraginaceae	Cordia sebestena L.	KT740817.1	16670	194	331	4.86E-87	97.423

Table S8. Continued.

Family/ Order	Interpreted Taxon	Accession #	Node	Length	Bit score	Evalue	%ID
Boraginaceae	Myriopus maculatus (Jacq.)Feul.	EU599648.1	33028	276	466	1.52E-127	97.101
Boraginaceae	Wigandia urens (R & Pav.) Kunth	KF158147.1	196627	161	291	5.14E-75	99.379
Brassicaceae	Raphanus raphanistrum L.	LR778315.1	102109	241	440	7.90E-120	99.585
Brassicaceae	Raphanus raphanistrum L.	KX545461.1	196090	188	337	7.94E-89	98.936
Burseraceae	Bursera sp.	KX426380.1	3429	471	859	0	99.575
Burseraceae	Bursera arborea (Rose) L.Riley	KM219804.1	643	688	1232	0	98.983
Burseraceae	Bursera simaruba L.	EU002206.1	7661	369	665	0	99.187
Burseraceae	Bursera simaruba L.	GU246028.1	2355	525	970	0	100
Burseraceae	Bursera simaruba L.	KJ773325.1	76920	252	466	3.00E-127	100
Burseraceae	Bursera simaruba L.	EU002206.1	12032	273	468	5.03E-128	97.802
Burseraceae	Bursera simaruba L.	EU002206.1	193491	206	364	4.07E-97	98.544
Burseraceae	Bursera sp.	KX522674.1	179616	159	283	8.53E-73	98.742
Burseraceae	Burseraceae	KY085915.1	196868	151	279	1.04E-71	100
Burseraceae	Burseraceae	GU246023.1	13867	240	444	6.08E-121	100
Burseraceae	Burseraceae	KF224981.1	6882	381	682	0	98.95
Burseraceae	Burseraceae	KU176163.1	11090	331	599	1.75E-167	99.396
Cannabaceae	Celtis sp.	AY257535.1	195269	192	327	5.34E-86	97.396
Combretaceae	Terminalia catappa L.	MH767792.1	117387	235	435	3.57E-118	100
Cyrillaceae	Cyrillaceae	KU350163.1	3047	173	309	5.07E-80	98.844
Fabaceae	Acacia	LN885243.1	196678	159	289	1.83E-74	99.371
Fabaceae	Caesalpinia pulcherrima (L.) Sw.	Z70153.1	13425	312	577	7.74E-161	100
Fabaceae	Caesalpinia pulcherrima (L.) Sw.	EU361906.1	7828	367	628	2.51E-176	97.548
Fabaceae	Caesalpinia sp.	KX373109.1	40013	271	490	8.85E-135	99.262
Fabaceae	Coursetia caribaea (Jacq.) Lavin	AF155814.1	15	540	992	0	99.815
Fabaceae	Delonix regia (Bojer) Raf.	NC_047368.1	77123	251	433	1.36E-117	98.008
Fabaceae	Fabaceae	Z70164.1	18793	288	521	3.36E-144	99.306
Fabaceae	Fabaceae	AF142718.1	3599	358	617	5.29E-173	97.765
Fabaceae	Fabaceae	AY650278.1	8	1353	2370	0	98.374
Fabaceae	Fabaceae	KJ468102.1	21	1350	2283	0	97.185
Fabaceae	Fabaceae	KJ468102.1	1105	401	680	0	97.257
Fabaceae	Fabaceae	KJ468102.1	7	1432	2483	0	97.975
Fabaceae	Fabaceae	KJ468102.1	1716	576	966	0	97.049
Fabaceae	Fabaceae	AF020471.1	196869	151	268	2.26E-68	98.675
Fabaceae	Gliricidia sepium (Jacq.) Steud.	LN552216.1	421	182	320	3.57E-83	98.352
Fabaceae	Gliricidia sepium (Jacq.) Steud.	KX057873.1	38	647	1195	0	100
Fabaceae	Gliricidia sepium (Jacq.) Steud.	AF400138.1	237	611	1123	0	99.836
Fabaceae	Haematoxylum brasiletto H.Karst.	NC_045040.1	1197	458	797	0	98.035

Table S8. Continued.

Family/ Order	Interpreted Taxon	Accession #	Node	Length	Bit score	Evalue	%ID
Fabaceae	Lonchocarpus sp.	JX506612.1	35652	274	484	8.00E-133	98.54
Fabaceae	Lonchocarpus guatemalensis Be.	KJ411669.1	10734	334	612	2.28E-171	99.701
Fabaceae	Lonchocarpus minimiflorus D.Sm.	JQ591874.1	174137	213	394	5.40E-106	100
Fabaceae	Lonchocarpus santarosanus DS	JX506613.1	65	1063	1916	0	99.153
Fabaceae	Fabaceae		219	380	664	0	98.158
Fabaceae	Phaseolus vulgaris L.	AC254328.1	7447	373	667	0	98.928
Fabaceae	Phaseolus vulgaris L.	EU196765.1	15984	293	542	2.66E-150	100
Lauraceae	Lauraceae	KT833081.1	2902	373	689	0	100
Lauraceae	Litsea sp.	KU382356.1	7327	374	684	0	99.733
Lauraceae	Persea americana Mill.	KX437771.1	13	1206	2915	0	99.44
Lauraceae	Persea americana Mill.	KX437771.1	68	1062	1940	0	99.623
Lauraceae	Persea americana Mill.	KX437771.1	2287	532	963	0	99.436
Lauraceae	Persea americana Mill.	KX437771.1	9707	344	636	1.40E-178	100
Lauraceae	Persea americana Mill.	KX509877.1	467	753	1264	0	97.344
Lauraceae	Persea americana Mill.	KX437771.1	4411	341	619	1.39E-173	99.413
Lauraceae	Persea americana Mill.	KX437771.1	4378	437	797	0	99.542
Lauraceae	Persea americana Mill.	KX437771.1	20	1261	2307	0	99.683
Lauraceae	Persea americana Mill.	KX437771.1	104	900	1652	0	99.778
Lauraceae	Persea americana Mill.	KX437771.1	1	2610	4815	0	99.962
Lauraceae	Persea americana Mill.	KX437771.1	437	723	1245	0	97.787
Lauraceae	Persea americana Mill.	KX437771.1	196619	161	298	3.10E-77	100
Lauraceae	Persea americana Mill.	KX437771.1	196592	162	300	8.68E-78	100
Lauraceae	Persea americana Mill.	KX437771.1	1356	596	1101	0	100
Lecythidaceae	Grias cauliflora L.	MF359952.1	90188	246	427	6.30E-116	97.967
Loranthaceae	Loranthaceae	JQ933439.1	41	1087	1903	0	98.252
Loranthaceae	Loranthaceae	HM010434.1	3206	460	821	0	98.913
Loranthaceae	Passovia pyrifolia (Kunth) Tiegh.	EU544448.1	12302	284	483	1.74E-132	97.535
Loranthaceae	Struthanthus oerstedii (Oliv.)St.	EU544457.1	74641	247	435	3.89E-118	98.381
Malvaceae	Guazuma ulmifolia Lam.	AF287918.1	127152	231	424	7.57E-115	99.567
Malvaceae	Hibiscus rosa-sinensis L.	AY328142.1	15065	299	529	2.09E-146	98.662
Malvaceae	Malvaceae	KY085914.1	9622	345	638	3.90E-179	100
Malvaceae	Malvaceae	AM235027.1	28228	279	483	1.53E-132	97.849
Malvaceae	Malvastrum coromandelianum L.	MK860037.1	28196	276	510	1.00E-140	99.64
Malvaceae	Malvastrum coromandelianum L.	MK860037.1	195982	195	350	2.00E-92	98.97
Malvaceae	Malvastrum coromandelianum L.	MK860037.1	9927	342	616	3.00E-172	99.12
Malvaceae	Malvastrum coromandelianum L.	MK860037.1	12341	321	577	2.00E-160	97.07
Malvaceae	Malvastrum coromandelianum L.	MK860037.1	196036	192	331	3.77E-87	97.917
Malvaceae	Malvastrum coromandelianum L.	MK860037.1	6264	236	399	2.30E-107	97.458
Malvaceae	Malvastrum coromandelianum L.	MK860037.1	40	1154	1993	0	97.834

Table S8. Continued.

Family/ Order	Interpreted Taxon	Accession #	Node	Length	Bit score	Evalue	%ID
Malvaceae	Malvastrum coromandelianum L.	MK860037.1	16502	293	525	2.65E-145	98.976
Malvaceae	Malvastrum coromandelianum L.	MK860037.1	3177	482	863	0	98.963
Malvaceae	Sida rhombifolia L.	KT966997.1	4057	270	494	1.19E-135	99.63
Meliaceae	Cedrela odorata L.	NC_037251.1	10990	324	577	8.27E-161	98.765
Meliaceae	Cedrela odorata L.	NC_037251.1	182362	210	377	5.35E-101	99.048
Moraceae	Brosimum alicastrum Sw.	GQ981947.1	33971	275	508	2.48E-140	100
Moraceae	Brosimum alicastrum Sw.	AF500373.1	2540	508	939	0	100
Moraceae	Brosimum alicastrum Sw.	AF500373.1	19743	287	531	5.56E-147	100
Moraceae	Brosimum guianense(Aubl.) Hub.	FJ039163.1	196400	171	316	9.22E-83	100
Moraceae	Brosimum lactescens (Moore)Ber	AY289329.1	7614	370	667	0	99.189
Moraceae	Castilla sp.	AY289322.1	6397	389	691	0	98.715
Moraceae	Maclura tinctoria (L.) D.Don	MG718980.1	43304	269	497	5.24E-137	100
Moraceae	Maclura tinctoria (L.) D.Don	KU855586.1	196303	176	315	3.43E-82	98.864
Moraceae	Maclura tinctoria (L.) D.Don	KU855589.1	276	847	1454	0	97.757
Moraceae	Moraceae	KU853143.1	196658	157	279	1.11E-71	98.726
Moraceae	Moraceae	HG963667.1	15956	208	385	4.75E-103	100
Moraceae	Moraceae	FJ037932.1	1506	591	1070	0	99.323
Moraceae	Trophis sp.	NC047182.1	109534	238	424	2.00E-114	99.15
Musaceae	Musa ornata Roxb.	NC_042874.1	13039	315	560	7.88E-156	98.73
Musaceae	Musa sp.	KY753133.1	14315	306	566	1.64E-157	100
Piperaceae	Piper aduncum Vell.	AY572252.1	725	703	1258	0	99.004
Piperaceae	Piper aduncum Vell.	AY572252.1	29610	278	508	2.51E-140	99.64
Piperaceae	Piper amalago L.	DQ868703.1	4410	395	713	0	99.241
Piperaceae	Piper amalago L.	DQ868703.1	1514	306	560	1.55E-155	99.673
Piperaceae	Piper auritum L.	KY085906.1	131	813	1483	0	99.631
Piperaceae	Piper auritum L.	KY085906.1	12502	318	555	3.72E-154	98.113
Piperaceae	Piper auritum L.	KY085906.1	70414	255	460	6.47E-126	99.216
Piperaceae	Piper auritum L.	KY085906.1	12632	318	544	8.02E-151	97.484
Piperaceae	Piper auritum L.	KY085906.1	2	1939	3386	0	97.937
Piperaceae	Piper auritum L.	KY085906.1	3	2179	3701	0	97.384
Piperaceae	Piper auritum L.	KY085906.1	1970	552	977	0	98.732
Piperaceae	Piper sp.	KY085899.1	9708	344	625	3.03E-175	99.419
Piperaceae	Piper umbellatum L.	EU519800.1	50412	265	490	8.63E-135	100
Poaceae	Dactyloctenium aegyptium (L.)Wi.	KY432807.1	163814	211	357	6.96E-95	97.63
Poaceae	Poaceae	KX507245.1	14	853	1535	0	99.179
Poaceae	Poaceae	MK348606.1	2502	516	874	0	97.287
Poaceae	Zea mays L.	MK348606.1	5	1963	3626	0	100
Poaceae	Zea mays L.	BT016655.1	46	871	1572	0	99.311

Table S8. Continued.

Family/ Order	Interpreted Taxon	Accession #	Node	Length	Bit score	Evalue	%ID
Poaceae	Zea mays L.	KP966117.1	4053	388	710	0	99.742
Poaceae	Zea mays L.	MK348606.1	22	1147	2119	0	100
Poaceae	Zea mays L.	KF241981.1	16	1392	2571	0	100
Poaceae	Zea sp.	MK348606.1	302	754	1386	0	99.867
Poaceae	Zea sp.	KP966116.1	142	870	1576	0	99.425
Pteridaceae	Adiantum capillus-veneris L.	AY178864.1	153571	221	387	9.43E-104	98.19
Rubiaceae	Deppea grandiflora Schltdl.	KY378675.1	1293	383	647	0	97.128
Rubiaceae	Deppea grandiflora Schltdl.	KY378675.1	18807	288	494	7.33E-136	97.569
Rubiaceae	Deppea grandiflora Schltdl.	KX426215.1	187	917	1559	0	97.492
Rubiaceae	Deppea grandiflora Schltdl.	KY378675.1	806	641	1118	0	98.128
Rubiaceae	Hamelia patens	GQ852219.1	12625	319	571	3.68E-159	99.06
Rubiaceae	Hamelia patens Jacq.	MF348493.1	12623	272	503	1.36E-138	100
Rubiaceae	Hamelia patens Jacq.	GQ982007.1	668	715	1321	0	100
Rubiaceae	Hamelia patens Jacq.	KX910860.1	3650	462	837	0	99.351
Rubiaceae	Hamelia patens Jacq.	GQ852219.1	1471	594	1086	0	99.663
Rubiaceae	Hamelia sp.	AM117361.1	1985	550	1005	0	99.636
Rubiaceae	Hamelia sp.	X83641.1	196047	191	346	1.34E-91	99.476
Rubiaceae	Hamelia sp.	X83641.1	196194	182	337	7.64E-89	100
Rubiaceae	Rogiera amoena Planch.	KY785284.1	329	594	1018	0	97.643
Rutaceae	Citrus sp.	KJ865401.1	10185	308	553	1.29E-153	99.026
Rutaceae	Citrus sp.	KJ865401.1	10762	309	516	1.84E-142	97.087
Rutaceae	Citrus aurantium L.	MT106672.1	8227	354	638	4.01E-179	99.153
Sapindaceae	Sapindus sp.	KY635881.1	33960	275	464	5.45E-127	97.091
Sapotaceae	Manilkara zapota (L.) P.Royen	HF542846.1	420	717	1315	0	99.721
Sapotaceae	Manilkara zapota (L.) P.Royen	MN295595.1	6263	391	712	0	99.488
Sapotaceae	Manilkara zapota (L.) P.Royen	MN295595.1	171378	214	390	7.02E-105	99.533
Sapotaceae	Manilkara zapota (L.) P.Royen	MN295595.1	24	1315	2209	0	97.034
Sapotaceae	Pouteria campechiana (Kunth) Baehni	KX426215.1	1127	512	918	0	99.023
Sapotaceae	Pouteria campechiana (Kunth) Baehni	KX426215.1	2947	493	861	0	98.174
Sapotaceae	Pouteria campechiana (Kunth) Baehni	KX426215.1	1897	557	987	0	98.743
Sapotaceae	Pouteria campechiana (Kunth) Baehni	KX426215.1	2388	520	961	0	100
Sapotaceae	Pouteria campechiana (Kunth)Baehni	KX426215.1	3913	365	625	3.58E-175	97.534
Sapotaceae	Pouteria sp.	JQ626061.1	9908	342	621	3.89E-174	99.415
Sapotaceae	Sapotaceae	KX426215.1	1922	554	990	0	98.917
Sapotaceae	Sideroxylon foetidissimum Jacq.	KJ773891.1	104546	240	427	6.12E-116	98.75

Table S8. Continued.

Family/ Order	Interpreted Taxon	Accession #	Node	Length	Bit score	Evalue	%ID
Saxifragaceae	Saxifragales	KY412195.1	94845	244	435	3.73E-118	98.77
Solanaceae	Brugmansia sp.	HG738853.1	16134	274	501	4.50E-138	99.635
Solanaceae	Iochroma sp.	KU310932.1	5090	414	713	0	97.826
Solanaceae	Lycianthes pauciflora (Vahl) Bitter	JN661833.1	6317	390	715	0	99.744
Solanaceae	Physalis angulata Lam.	MH045574.1	52260	264	483	1.44E-132	99.621
Solanaceae	Solanaceae	KU199713.1	132014	229	401	3.51E-108	98.253
Solanaceae	Solanaceae	KU199713.1	187704	209	374	6.84E-100	99.043
Solanaceae	Solanaceae	KJ773743.1	196029	192	355	2.25E-94	100
Solanaceae	Solanum anceps Ruiz & Pav.	MK860037.1	354	283	523	9.16E-145	100
Solanaceae	Solanum demissum Lindl.	NC_041552.1	847	360	623	1.57E-174	98.056
Solanaceae	Solanum erianthum D.Don	KP093239.1	9530	346	604	3.97E-169	98.266
Solanaceae	Solanum erianthum D.Don	MH718332.1	3887	354	654	0	100
Solanaceae	Solanum erianthum D.Don	KJ773910.1	196265	178	329	1.24E-86	100
Solanaceae	Solanum erianthum D.Don	MH837795.1	1726	522	948	0	99.425
Solanaceae	Solanum erianthum D.Don	MH050096.1	2710	504	913	0	99.405
Solanaceae	Solanum lepidotum H & B	GQ982099.1	335	823	1498	0	99.514
Solanaceae	Solanum lignescens Fernald	JN661827.1	3150	483	833	0	97.93
Solanaceae	Solanum lycopersicum L.	KP117024.1	63	878	1611	0	99.772
Solanaceae	Solanum phaseoloides Pol.	HQ856060.1	5247	415	706	0	97.59
Solanaceae	Solanum rovirosanum Donn.Sm.	DQ180437.1	196847	152	281	2.92E-72	100
Solanaceae	Solanum sp.	U47415.1	1312	612	1114	0	99.51
Solanaceae	Solanum sp.	HM850366.1	38431	269	497	5.31E-137	100
Solanaceae	Solanum sp.	KT375308.1	23	1226	2170	0	98.613
Solanaceae	Solanum sp.	MF159407.1	4191	443	791	0	98.871
Solanaceae	Solanum sp.	EF439039.1	190388	207	372	2.44E-99	99.034
Solanaceae	Solanum stramoniifolium Benth.	MN218087.1	50649	265	451	4.07E-123	97.358
Urticaceae	Urticaceae	KY611451.1	181121	211	363	1.51E-96	97.63
Viburnaceae	Viburnum sp.	NC_048464.1	288	845	1487	0	98.462

Table S9. Plant species and marker sequences (Genbank) used to create the genetic probe.

Species	Family	ycf1	trnT-trnL	trnL-trnF	rpl10	rbcL	psbA-trnH	ndhF	matK
Agave americana L.	Agavaceae				GQ402511.1				JX903544.1
Agave sisalana Perrine ex. Engelm.	Agavaceae					GU135234.1	GU135405.2		GU135070.1
Amaranthus hybridus L.	Amaranthaceae					MF135386.1	MF143703.1		MF159449.1
Amaranthus hypochondriacus L.	Amaranthaceae					X51964.1	MF143751.1		MG685144.1
Anacardium occidentale L.	Anacardiaceae			AY594497.1		AY462008.1	KR075990.1	KF664342.1	AY594459.1
Spondias purpurea L.	Anacardiaceae			KR081868.1		KU559308.1	KJ026821.1		KP774612.1
Annona glabra L.	Annonaceae	GU937365.1							
Annona muricata L.	Annonaceae		DQ861648.1	AY145352.1		AY743440.1	AY841428.1	EF179282.1	AF543722.1
Xanthosoma sagittifolium (L.) Schott	Araceae			AY555175.1		L10246.2			EU886500.1
Acrocomia aculeata (Jacq.) Lodd.	Arecaceae			HQ265759.1		AY044625.1	KJ426591.1	AY044555.1	AM114639.1
Attalea cohune Mart.	Arecaceae	KU323514.1							
Bactris gasipaes Kunth	Arecaceae			DQ445909.1		GQ981678.1		EU004890.1	HQ265554.1
Bactris major Jacq.	Arecaceae					AM110214.1	HG963669.1		HQ265556.1
Chamaedorea tepejilote Liebm.	Arecaceae			AM497773.1		GQ981699.1	GQ982181.1	DQ273108.1	DQ178691.1
Elaeis oleifera (Kunth) Cortés	Arecaceae			HQ265802.1		AY012509.1	GQ982210.1	AY044562.1	HQ265568.1
Helianthus annuus L.	Asteraceae		AY215931.1	KM385516.1		L13929.1	JF321291.1	AB530934.1	AY215805.1
Crescentia alata Kunth	Bignoniaceae							FJ887857.1	
Crescentia cujete L.	Bignoniaceae					KJ082242.1	KJ426683.1		JQ587002.1
Bixa orellana L.	Bixaceae			FM179540.1		AF022128.1	HG963562.1	EU077547.1	FM179929.1
Ananas comosus (L.) Merr.	Bromeliaceae	KU096009.1		KU762973.1		L19977.1	AB331266.1	AY147766.1	KU258039.1
Brasenia schreberi J.F. Gmel.	Cabombaceae		AY145329.1	AM489713.1		KX527460.1	AB331296.1		AF092973.1
Cabomba aquatica Aubl.	Cabombaceae					JX100671.1	JX100499.1		
Opuntia ficus-indica (L.) Mill.	Cactaceae					FJ026615.1	FJ026613.1	JF787380.1	FN997314.1
*Opuntia guatemalensis*Britt & Rose	Cactaceae								JQ587177.1
Canna indica L.	Cannaceae			AM113702.1		HM849844.1	AB331269.1	FJ861155.1	AM114724.1
Carica papaya L.	Caricaceae			JX091823.1		JX091914.1	KC867738.1	AY483248.1	AY483221.1
Chenopodium berlandieri Moq.	Chenopodiaceae			HE577567.1		MG249740.1			HE855645.1
Ipomoea batatas (L.) Lam.	Convolvulaceae			AY101071.1		AY942199.1		AF130177.1	AJ429355.1
Cucurbita argyrosperma K. Koch	Cucrbitaceae			HQ438663.1		HQ438617.1			HQ438592.1
Cucurbita ficifolia Bouché	Cucrbitaceae			HQ438677.1		HQ438631.1			HQ438599.1
Cucurbita moschata Duchesne	Cucrbitaceae			HQ438660.1		HQ438614.1			HQ438603.1
Cucurbita pepo L.	Cucrbitaceae					L21938.1			HQ438611.1
Lagenaria siceraria (Molina) Standl.	Cucrbitaceae			DQ536771.1		DQ535825.1	GQ248323.1		DQ536694.1
Sechium edule (Jacq.) Sw.	Cucrbitaceae			DQ536861.1		AY862553.1	JN560308.1		DQ536727.1
Dioscorea trifida L.f.	Dioscoreaceae		D89681.1	D89683.1		KM877929.1	KR072380.1		KR072314.1
Manihot esculenta Crantz	Euphorbiaceae			EU518905.1		LT576833.1	KP692112.1		GU214863.1

Table S9. Continued.

Species	Family	ycf1	trnT-trnL	trnL-trnF	rpl10	rbcL	psbA-trnH	ndhF	matK
Canavalia cathartica Thouars	Fabaceae						GU396816.1		
Canavalia ensiformis (L.) DC.	Fabaceae			EU717354.1		U74238.1			KT751481.1
Indigofera suffruticosa Mill.	Fabaceae					KX119304.1	HG963791.1		AF142697.1
Pachyrhizus erosus (L.) Urb.	Fabaceae			EU717324.1		EU717260.1	GU396706.1		EU717401.1
Phaseolus acutifolius A.Gray	Fabaceae								DQ445952.1
Phaseolus coccineus L.	Fabaceae	CA910235.1	GQ411774.1	JQ041849.1		LT576851.1	JX495464.1		DQ445965.1
Phaseolus lunatus Billb. ex Beurl.	Fabaceae								DQ445985.1
Phaseolus vulgaris L.	Fabaceae	JZ715503.1	GQ411777.1	EU717342.1		KX119315.1	FJ951239.1		DQ450862.1
Persea americana Mill.	Lauraceae		JQ742021.1	GU250776.1		AY337727.1	JF966417.1	JQ437545.1	AJ247179.2
Byrsonima crassifolia (L.) Kunth	Malpighiaceae			AF350945.1		AB233898.1	GQ429122.1	AF351011.1	AF344535.1
Gossypium hirsutum L.	Malvaceae	AY800381.1	AF031434.1	HQ696725.1		M77700.1	HM437901.1	U55340.1	AY321158.1
Theobroma cacao L.	Malvaceae			HM488410.1		AF022125.1	MF348567.1	AF287916.1	AY321195.1
Calathea crotalifera S. Watson	Marantaceae					AY656112.1		AY656083.1	
Calathea lutea (Aubl.) Schult.	Marantaceae			JQ341231.1			GQ429113.1		JQ341348.1
Maranta arundinacea L.	Marantaceae			AY140384.1		JQ592612.1	HG963784.1		JQ341325.1
Brosimum alicastrum Sw.	Moraceae			AF501601.1		AF500346.1	HG963667.1	AY289328.1	GQ981947.1
Psidium guajava L.	Myrtaceae					KX527097.1	JQ279707.1		AB354958.1
Nymphaea ampla (Salsb.) DC.	Nymphaeaceae		AM422044.1	AM422044.1					
Vanilla planifolia Andrews	Orchidaceae	JN181530.1		AY557223.1		JN181479.1	MF348723.1		JN181462.1
Piper auritum Kunth	Piperaceae	NC_034697.1		EU519625.1		EF590560.1	EU581473.1	EU519715.1	DQ882205.1
Setaria macrostachya Kunth	Poaceae							EU741956.1	
Zea mays L.	Poaceae		EF541347.1	GQ870012.1	NM_001359379.1	MG226097.1	AF543684.1	U21985.1	X86563.2
Prunus serotina Ehrh.	Rosaceae		AM950169.1	JX414453.1		DQ006123.1	HQ596803.1	JQ776954.1	HQ235266.1
Manilkara zapota (L.) P.Royen	Sapotaceae	KP088426.1		DQ924309.1		EU980807.1	GU135342.2	AY230696.1	DQ924092.1
Pouteria campechiana (Kunth) Ba.	Sapotaceae			DQ344318.1					
Pouteria sapota (Jacq.)Mre & Strn.	Sapotaceae				GQ402492.1				
Capsicum annuum L.	Solanaceae			AY348966.1		KJ773334.1	JQ087869.1	DQ667527.1	EF537302.1
Datura stramonium L.	Solanaceae			EU580984.1				EU580875.1	KP756825.1
Nicotiana rustica L.	Solanaceae					MG221507.1		AJ585935.1	AB039992.1
Nicotiana tabacum L.	Solanaceae			FJ490822.1	NM_001324800.1	KC825342.1	FJ493313.1	L14953.1	KJ652184.1
Physalis philadelphica Lam.	Solanaceae			EU581045.1		MG222711.1		EU580929.1	EF438953.1
Solanum lycopersicum L.	Solanaceae		DQ180450.1	DQ180450.1	AB518477.1	L14403.1	GU562406.1	U08921.1	

Table S10. Results shown here are from the PicoGreen analysis of project samples submitted to RAPiD Genomics. Note that in the 6th column there are readings of DNA recorded from each sample. Eight archaeological samples and the Uaxactun garden sample contained enough DNA to proceed to whole genome amplification and library preparation.

Provenance	Op. #	Lot	Depth	Seq. Name	DNA (ng/µL)	Time Period
Corriental Reservoir	1	C	50cm	Tik2	0	Post Classic
Corriental Reservoir	1	C	70cm	Tik47	0	Terminal Postclassic
Corriental Reservoir	1	C	100cm	Tik3	0	Late Classic
Corriental Reservoir	1	C	210cm	Tik4	0	Early Classic
Corriental Reservoir	1	C	280cm	Tik5	0	Preclassic
Aguada de Terminos	5	F	50cm	Tik7	0	
Aguada de Terminos	5	F	100cm	Tik8	0	
Palace Reservoir	6	L	10 cm	Tik33	10.89192467	A horizon
Palace Reservoir	6	L	110 cm	Tik9 (WA08)	0.178947564	Postclassic
Palace Reservoir	6	L	170 cm	Tik10(WA01)	0.113304322	Terminal Classic/ Late Classic
Palace Reservoir	6	L	210 cm	Tik11	0	Late Classic
Palace Reservoir	6	L	215 cm	Tik49(WA07)	1.557455647	Early Preclassic
Perdido Reservoir	8	A	10cm	Tik34	2.489589684	A1 horizon
Perdido Reservoir	8	A	50cm	Tik31	1.363808083	plaster floor/clay (285BC)
Perdido Reservoir	8	A	70cm	Tik12	0	organic clay fill (390-180BC)
Perdido Reservoir	8	A	90cm	Tik50	0	Early Classic (AD350-540)
Perdido Reservoir	8	A	110cm	Tik13	0	Early Classic (AD350-540)
Perdido Reservoir	8	A	150cm	Tik15	0	subfloor (pre-Maya bajo)
Perdido Reservoir	8	A	170cm	Tik16	0	pre-Maya bajo soil
Temple Reservoir	7	C	55cm	Tik17	0	C1 horizon
Temple Reservoir	7	C	65cm	Tik18	0	C2 horizon
Temple Reservoir	7	C	75cm	Tik20	0	C3 horizon
Temple Reservoir	7	C	85cm	Tik21	0.12643297	C4 horizon (AD 680-890)
Temple Reservoir	7	C	95cm	Tik22	0	C5-6 horizon
Temple Reservoir	7	C	105cm	Tik23	0	C7 horizon
Temple Reservoir	7	C	115cm	Tik24(WA09)	0.694247014	Late Classic (C7 horizon)
Aguada Vaca de Monte	11		27cm	Tik 29	0	early historic
Aguada Vaca de Monte	11		32cm	Tik28	0	Postclassic
Aguada Vaca de Monte	11		37cm	Tik27	0	Late Classic
Uaxactun Home Garden	U	H	10 cm	Tik41(WA05)	2.62415833	Modern

SI References

1. Dunning, N. P., Beach, T. & Luzzadder-Beach, S. Kax and kol: collapse and resilience in lowland Maya civilization. *Proc. Natl. Acad. Sci. U.S.A.* **109**, 3652-3657 (2012).

2. Stephens, J. L. *Incidents of travel in Yucatan. Vol. 2* (Harper & Brothers,1843).

3. Bullard, Jr., W. R. Maya settlement pattern in northeastern Petén, Guatemala. *Am. Antiq.* **25**, 355-372 (1960).

4. Lundell, C. L. The vegetation of the Petén. Carnegie Institution of Washington Publication **478,** (1937).

5. Maler, T. *Península Yucatán*. Gebr Mann Verlag (1997).

6. Morley, S. G. The Inscriptions of Petén, Vol. 1. Carnegie Institution of Washington, Publication ***437*** (1938).

7. Dunning, N. P. Lords of the hills: ancient Maya settlement in the Puuc region, Mexico. *Monographs in World Archaeology* **15**. (Prehistory Press, 1992).

8. Harrison, P. D. The marvel of Maya engineering: water management at Tikal. *Expedition* **54**, 19-26 (2012).

9. Scarborough, V. L. & Gallopin, G. G. A water storage adaptation in the Maya Lowlands. *Science* **251**, 658-662 (1991).

10. Lentz, D. L. & Hockaday, B. Tikal timbers and temples: ancient Maya agroforestry and the end of time. *J. Archaeol. Sci.* **36,** 1342-1353 (2009).

11. Grazioso Sierra, L. & Scarborough, V. Control de agua por los antiguos Mayas: el sistema hidráulico de Tikal. *Contrib. N. World Archaeol.* **5**, 39-56 (2013).

12. Scarborough, V. L. & Grazioso Sierra, L. The evolution of an ancient waterworks system at Tikal in *Tikal: paleoecology of an ancient Maya city* (eds. Lentz, D. L., Dunning, N. P. & Scarborough, V. L.) 16-45 (Cambridge University Press, 2015).

13. Dunning, N. P. *et al.* Life on the edge: Tikal in a bajo landscape in *Tikal: paleoecology of an ancient Maya city* (eds. Lentz,D. L., Dunning, N. P. & Scarborough, V. L.) 95-123 (Cambridge University Press, 2015).

14. Lane, B., Scarborough, V. L. & Dunning, N. P. At the core of Tikal: terrestrial sediment sampling and water management in *Tikal: paleoecology of an ancient Maya city* (eds. Lentz,D. L., Dunning, N. P. & Scarborough, V. L.) 46-58 (Cambridge University Press, 2015).

15. Tankersley, K. B. *et al.* Evidence for volcanic ash fall in the Maya lowlands from a reservoir at Tikal, Guatemala. *J. Archaeol. Sci.* **38**, 2925-2938 (2011).

16. Tankersley, K. B. *et al.* “Fire and water: the archaeological significance of Tikal’s quaternary sediments.” in *Tikal: paleoecology of an ancient Maya city* (eds. Lentz,D. L., Dunning, N. P. & Scarborough, V. L.) 186-211 (Cambridge University Press, 2015).

17. Lentz, D. L. *et al.* Molecular genetic and geochemical assays reveal severe contamination of drinking water reservoirs at the ancient Maya city of Tikal. *Sci. Rep.* **10**,10316 (2020).

18. Scarborough, V. L., Connelly, R. & Ross, S. The prehispanic Maya reservoir system at Kinal, Petén, Guatemala. *Anc. Mesoam.* **5**, 97-106 (1994).

19. Tokovinine, A. Place and identity in Classic Maya narratives. *Studies in Pre-Columbian Art and Archaeology* ***37**.* (Dumbarton Oaks, 2013).

20. Scarborough, V. L. *et al*. Water and sustainable land use in an ancient tropical city: Tikal, Guatemala. *Proc. Natl. Acad. Sci. U.S.A.* **109**, 12408-12413 (2012).

21. Fulton, T.L. & Shapiro, B. Setting up an ancient DNA laboratory in Ancient DNA: methods and protocols, 2nd ed. (eds. B. Shapiro, et al.) 1-14 (Humana Press, 2019).

22. Jin, S., Kim, K.Y., Kim, M. S. & Park, C. An assessment of the taxonomic reliability of DNA barcode sequences in publicly available databases. *Algae* **35**, 293-301 (2020).

23. Jørgensen, T. *et al*. Islands in the ice: detecting past vegetation on Greenlandic nunataks using historical records and sedimentary ancient DNA meta-barcoding. *Mol. Ecol.* **21**, 1980-1988 (2012).

24. Sønstebø, J. H. *et al*. Using next-generation sequencing for molecular reconstruction of past Arctic vegetation and climate. *Mol. Ecol. Resour.* **10**, 1009-18 (2010).

25. Willerslev, E. *et al*. Fifty thousand years of Arctic vegetation and megafaunal diet. *Nature* **506**, 47–51 (2014).

26. Pearsall, D. M. Paleoethnobotany: a handbook of procedures. (Routledge, 2015).

27. Lentz, D. L. *et al.* Forests, fields, and the edge of sustainability at the ancient Maya city of Tikal. *Proc. Natl. Acad. Sci. U.S.A.* **111**, 18513–18518 (2014).

28. Lentz, D. L. *et al*. Agroforestry and agricultural practices of the ancient Maya at Tikal in *Tikal: paleoecology of an ancient Maya city*, (eds. Lentz, D. L., Dunning, N. P. & Scarborough, V. L.) 152-185 (Cambridge University Press, 2015).

29. Lentz, D. L. *et al.* Imperial resource management at the ancient Maya city of Tikal: A resilience model of sustainability and collapse. *J. Anthropol. Archaeol.* **52**, 113-122 (2018).

30. Hamilton, T. L., Peters, J. W., Skidmore, M. L. & Boyd, E. S. Molecular evidence for an active endogenous microbiome beneath glacial ice. *ISME* J. **7**, 1402-1412 (2013).

31. Epp, L., Zimmermann, H. & Leichsenring, K. Sampling and extraction of ancient DNA from sediments in *Ancient DNA: Methods and Protocols, B.* (eds. Shapiro, B. *et al*.) 31-44 (Springer, 2019).

32. Cooper, A. & Poinar, H. Ancient DNA: do it right or not at all. *Science* **289**, 5482 (2000).

33. Ahn, S.J., J. Costa & J. R. Emanuel. PicoGreen quantitation of DNA: effective evaluation of samples pre- or post-PCR. *Nucleic Acids Research* **24**, 2623–2625 (1996).

34. Li, H. & Durbin, R. Fast and accurate short read alignment with Burrows-Wheeler Transform. *Bioinformatics* **25,** 1754-60(2009).

35. Standley, P. C. *et al.* Flora of Guatemala. *Fieldiana*, Bot. **24** (1946-1977).

36. Royal Botanic Gardens, Kew, Online plant database. (http//:plantsoftheworldonline.org). 2020.

37. Lentz, D. L. Anthropocentric food webs in the Precolumbian Americas in *Imperfect Balance: Landscape Transformations in the Precolumbian Americas* (ed. Lentz, D.L.) 89-120 (Columbia University Press, 2000).

38. Moholy-Nagy, H. The artifacts of Tikal: utilitarian artifacts and unworked material, Tikal Report No. 27(B). University of Pennsylvania Museum of Archaeology and Anthropology (2003).

39. Pohl, M., Pope, K. & Jones, J. Base agrícola de la civilización Maya de las tierras bajas in XIII Simposio de Investigaciones Arqueológicas en Guatemala (eds. Laporte, J.P. Escobedo, H. Arroyo, B. Susnávar, A. C.) 258-267 (Museo Nacional de Arqueología y Etnología, 2000).

40. Lentz, D. L., Dunning, N. P. & Scarborough V. L. (eds.) *Tikal: Paleoecology of an Ancient Maya City* (Cambridge University Press, 2015).
